# Exploring the Charge-Transport and Optical Characteristics of Organic Doublet Radicals: A Theoretical and Experimental Study with Photovoltaic Applications

**DOI:** 10.1021/acsami.4c08524

**Published:** 2024-07-25

**Authors:** Mariia Stanitska, Rasa Keruckiene, Gjergji Sini, Dmytro Volyniuk, Arunas Marsalka, Zhong-En Shi, Chung-Ming Liu, Yan-Ru Lin, Chih-Ping Chen, Juozas V. Grazulevicius

**Affiliations:** † Department of Polymer Chemistry and Technology, Kaunas University of Technology, K. Barsausko St. 59, LT-50254 Kaunas, Lithuania; ‡ Laboratoire de Physicochimie des Polymères et des Interfaces, CY Paris Cergy Université, EA 2528, 5 mail Gay-Lussac, Cergy-Pontoise, Cedex 95031, France; § Faculty of Physics, Vilnius University, Sauletekio st. 9-3, LT-10222 Vilnius, Lithuania; ∥ Department of Materials Engineering, Ming Chi University of Technology, 84 Gunjuan Road, Taishan, New Taipei City 24301, Taiwan, Republic of China; ⊥ College of Engineering, Chang Gung University, Taoyuan City 33302, Taiwan, Republic of China

**Keywords:** radical, PSC, blue emission, dimethylacridan, carbazole

## Abstract

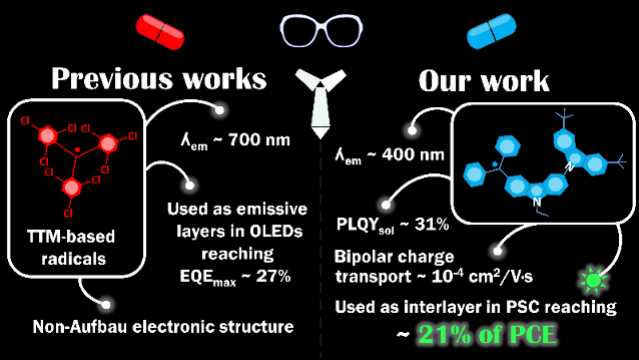

Herein, we present a series of stable radicals containing a trityl carbon-centered radical moiety exhibiting interesting properties. The radicals demonstrate the most blue-shifted anti-Kasha doublet emission reported so far with high color purity (full width at half-maximum of 46 nm) and relatively high photoluminescence quantum yields of deoxygenated toluene solutions reaching 31%. The stable radicals demonstrate equilibrated bipolar charge transport with charge mobility values reaching 10^–4^ cm^2^/V·s at high electric fields. The experimental results in combination with the results of TD-DFT calculations confirm that the blue emission of radicals violates the Kasha rule and originates from higher excited states, whereas the bipolar charge transport properties are found to stem from the particularity of radicals to involve the same molecular orbital(s) in electron and hole transport. The radicals act as the efficient materials for interlayers, passivating interfacial defects and enhancing charge extraction in PSCs. Consequently, this leads to outstanding performance of PSC, with power conversion efficiency surpassing 21%, accompanied by a remarkable increase in open-circuit voltage and exceptional stability.

## Introduction

The development of efficient organic semiconductors is a prerequisite for the fabrication of highly efficient optoelectronic devices.^1^ Stable radicals find applications in various optoelectronic devices, including organic light-emitting diodes (OLEDs),^2^ organic photovoltaics (OPVs),^3^ and organic field-effect transistors (OFETs).^4^ In OLEDs, stable radicals serve as emitters, leveraging their unique electronic structures for efficient doublet emission, spanning from red to deep-red colors and potentially extending to green and blue with further advancements.^5−7^ This emission occurs through the photoexcitation of the doublet-ground state, generating fluorescence with short emission lifetimes, thereby boosting device efficiency.^8^ Stable radicals address challenges faced by conventional emitters, such as low photostability and inefficient photoluminescence.^9^ Moreover, they exhibit bipolar charge transport properties, facilitating efficient charge carrier movement within the device.^10−12^

Perovskite solar cells (PSCs) have recently attracted significant research interest due to their tunable bandgap, high optical absorption, and impressive power conversion efficiency (PCE). Single-junction PSCs have already surpassed PCE of 25%.^13,14^ While inverted PSCs still lag behind their regular single-junction counterparts in terms of PCE, there has been substantial growth in the past three years, primarily due to the development monolayers of carbazole-based self-assembled compounds for use as hole transport layers (HTLs) or hole-selective layers (HSLs) at the anode contact in PSCs.^15−17^ Regarding these outstanding inverted PSCs, rapid advancements in charge transport layers (CTLs) and their interfacial engineering,^18,19^ along with passivation strategies,^20−22^ have enabled the realization of high-performance and ambient-stable perovskite-based devices. Nevertheless, defects frequently appear at the interface between the CTL and the perovskite layer, leading to nonradiative recombination of charge carriers and reduced device efficiency. To address this issue, many researchers have attempted to passivate these defects by introducing a buried layer or interlayer between the CTL and the perovskite layer.^23−28^ Various radicals have been developed as perovskite additives, including TEMPO,^29^ DMBI-2-Th-I,^30^ and TTM.^31^ These radicals have demonstrated varying levels of effectiveness in passivating PSCs, minimizing trap density, and reducing recombination within the devices. However, the use of free radicals as interlayers in PSCs is relatively uncommon. In OPVs and OFETs, stable radicals contribute to improved charge generation, transport, and injection, enhancing device performance and enabling novel applications in energy harvesting and electronic circuitry. Thus, the incorporation of stable radicals in optoelectronic devices broadens the scope for advancements in lighting, energy conversion, displays, and beyond.

In this context, the successful employment of such materials in optoelectronics needs the development of innovative synthesis methods of new charge-transporting and/or luminescent radicals. The difficulty in the development of such materials is the limited radical samples available. One way is introduction of steric hindrance inducing groups to the radical moiety.^5^ The incorporation of a sp2 alkyl group adjacent to the trivalent radical carbon can lower the π-delocalization.^32^ Three series of widely reported room temperature luminescent stable radicals, perchlorotriphenyl methyl (PTM) radical derivatives,^33^ tris(2,4,6-trichlorophenyl) methyl (TTM) radical derivatives,^33^ and pyridyl-containing triarylmethyl radicals (PyBTM),^33^ have the central radical structure surrounded by three benzene units with chlorine atoms. Such design strategy to induce the steric hindrance can enhance the stability of organic radicals in organic optoelectronics.^34,35^

Despite the aforementioned achievements, stable luminescent and/or charge transporting radicals and doublet-emission based devices are still in their infancy and strongly depend on the innovative approaches for the development of new stable radical based organic electronically active compounds. Here we present a new series of stable radicals, which were synthesized taking into account the structural advantages induced by steric effect of alkylation. An effective approach for shielding the radical site by using methyl substituents prohibited dimerization. It induced the spin delocalization effect that is beneficial to narrow the energy gap and decrease the entire energy as well as the radical site reactivity. Subsequently, we introduced radicals for passivation between the HSL and the perovskite layer.

## Results and Discussion

### Synthesis and Thermal Properties

**Scheme 1:**
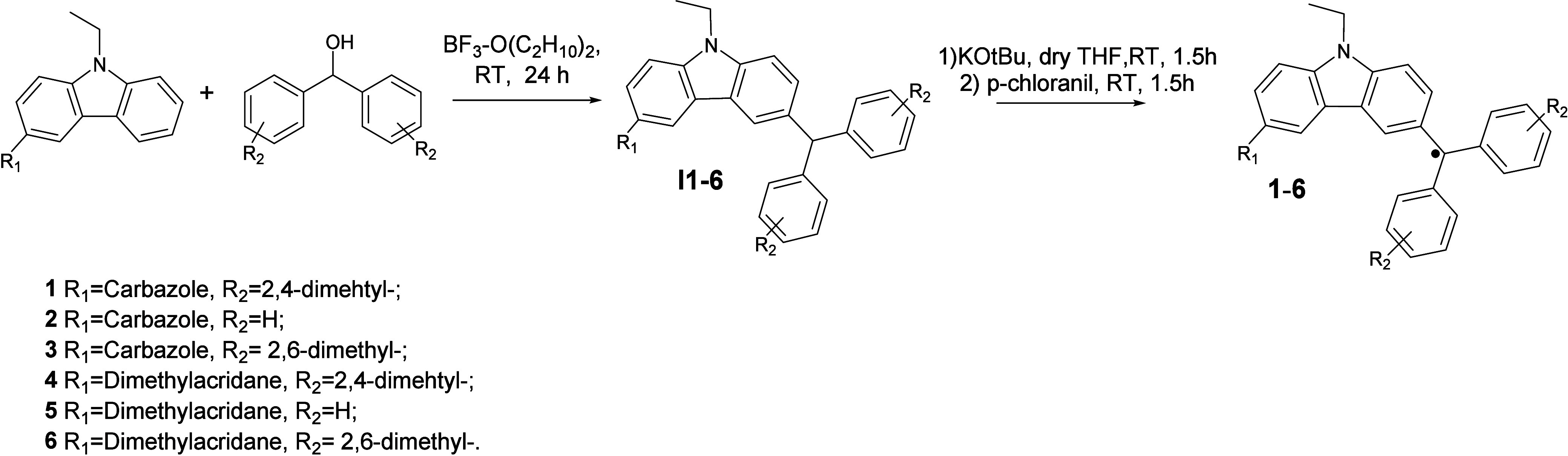
General Scheme for Obtaining Compounds **1**–**6**

Bipolar electroactive compounds containing a trityl carbon-centered stable radical moiety were synthesized via Friedel–Crafts alkylation reaction.^36^ Aromatic amine was alkylated by the reaction with aromatic alcohol in the presence of Lewis acid as catalyst. The following dehydrogenation was performed by adding *p*-chloranyl to obtain stable radical in medium yields (∼40%). The general synthesis schemes of compounds **1**–**6** are presented (Scheme 1).

The structures of the synthesized intermediate compounds were confirmed by ^1^H NMR, FT-IR spectroscopies, and mass spectrometer. The electron paramagnetic resonance (EPR) method was used to approve the radical form of target compounds (Figure S1). Analysis of the spectra is given in SI.

To estimate the possibilities of the solid state applications of the stable radicals, their samples were studied by thermogravimetric analysis (TGA) and differential scanning calorimetry (DSC) (Figure S2). The main results of those thermal characterizations of compounds **1**–**6** are collected in Table 1.

**Table 1:** Thermal Characteristics of Stable Radicals **1**–**6**

Compound^a^	1	2	3	4	5	6
T_g_, °C	127	117	90	83	105	100
T_d-10%_, °C	385	355	318	393	384	320

a T_g_ – glass transition temperature; T_d-10%_ – 10% weight loss temperature.

The stable radicals **1**–**6** were characterized by relatively high decomposition temperatures. Their temperatures of 10% weight loss (T_d-10%_,) exceeded 320 °C (Table 1). Glass-transition temperatures (T_g_) higher than 100 °C were obtained for the compounds **1**–**6** during the second heating scans displaying that they form molecular glasses.

### Structural and Electronic Properties

**Figure 1:**
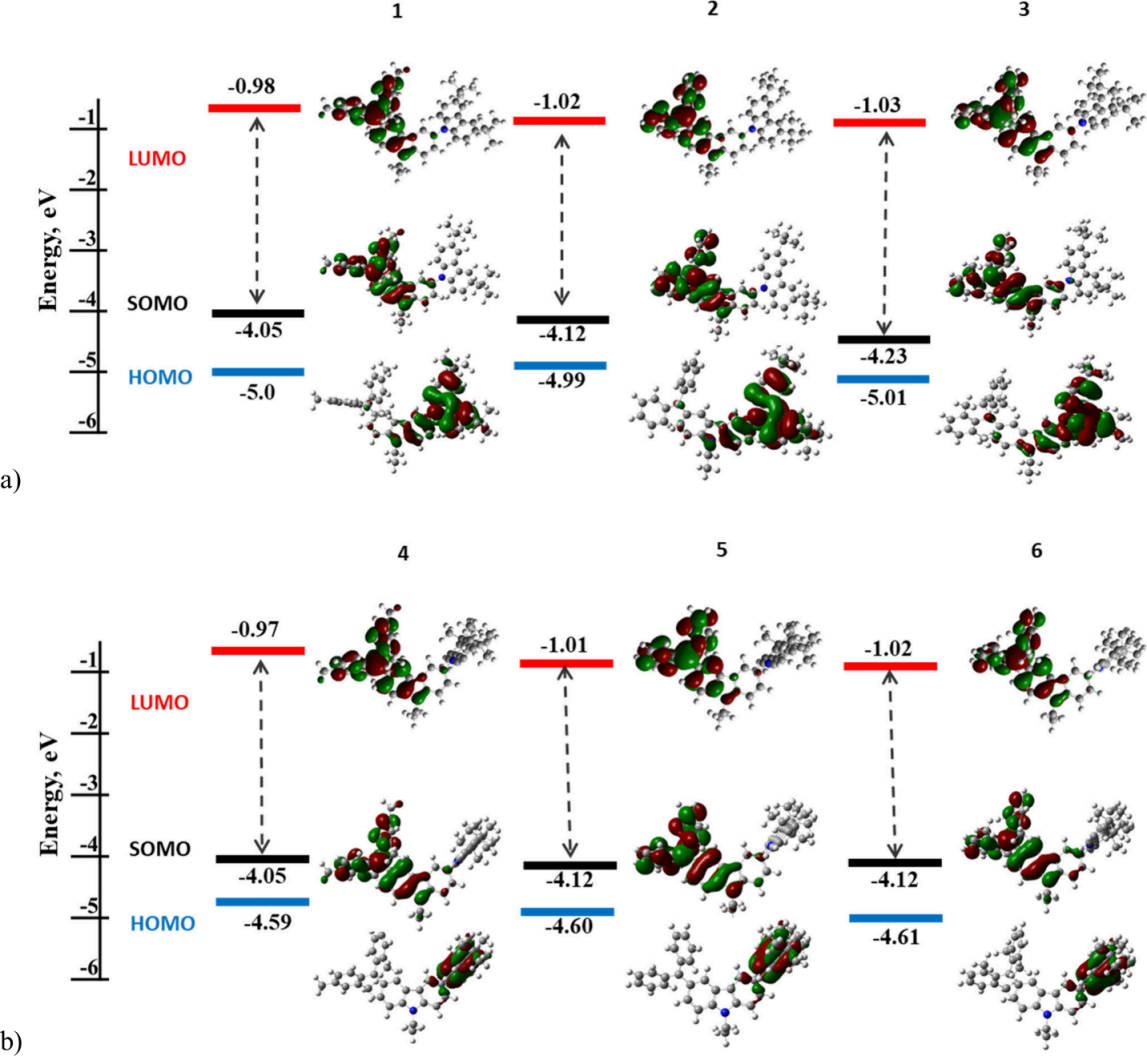
Molecular orbitals distribution (α) of carbazole-containing radicals **1**–**3** (a) and dimethylacridan-containing radicals **4**–**6** (b) at the ground state level (calculated in vacuum).

The geometries of the compounds were optimized at the B3LYP/6-31+G** level and are shown in Figure 1a. The dihedral angles between the central carbazole group and the donor lateral moiety range 65–75° for compounds **1**–**3** (carbazole donor group) and are practically 90° for compounds **4**–**6** (dimethylacridan donor moiety). This difference is translated in a HOMO α delocalization over both the central and the lateral carbazoles for compounds **1**–**3**, whereas exclusive localization over dimethylacridan moiety can be observed in the case of compounds **4**–**6**, see Figure 1b.

### Electrochemical and Photoelectrical Properties

To estimate electrochemical stability of the investigated compounds, cyclic voltammetry measurements (CV) have been performed (Figure S3). The obtained ionization potential (IP^CV^), electron affinity (EA^CV^), and energy gap (E_G_^CV^) values are presented in Table 2.

**Table 2:** Electrochemical Characteristics of Stable Radicals **1**–**6**^a^

Compound	IP^CV^, eV	EA^CV^, eV	E_G_^CV^, eV	IP_PE_, eV	HOMO	LUMO
**1**	5.45	4.43	1.02	5.76	–5.00	–0.98
**2**	5.45	–*	–	5.35	–4.99	–1.02
**3**	5.62	4.47	1.15	5.76	–5.01	–1.03
**4**	5.19	4.28	0.91	5.40	–4.59	–0.97
**5**	5.25	4.38	0.87	5.35	–4.60	–1.01
**6**	5.29	4.40	0.89	5.41	–4.61	–1.02

a IP_CV_ is ionization energy estimated by CV as IP_CV_ = *E*_*onset*__ox vsFc_ + 5.1 eV. *E*_g_^CV^ is electrochemical band gap estimated estimated by CV as *E*_g_^CV^ = IP_CV_ – EA_CV_. EA_CV_ is electron affinity estimated as EA_CV_ = *E*_*onset*__red vsFc_ – 5.1 eV. IP_PE_ is ionization potential estimated from electron emission in air spectra. ∗ – reduction was not detected during the CV measurement (see Figure S3).

All the precursors are electrochemically stable as they demonstrate two reversible oxidation peaks that indicate formation of radical cations (the first peak) and diradical dications (the second peak) (Figure S2). Ionization potential and electron affinity values were estimated from the ferrocene standard potential values. IP^CV^ values of the stable radicals depend on the electron-donating nature of dimethylacridan and carbazole substituents attached at the carbazole core. Dimethylacridan-containing radicals **4**–**6** have lower IP^CV^ values indicating better electron donating abilities. Whereas EA^CV^ values were found to be very similar indicating the influence of radical acceptor. Materials that present EA^CV^ values higher than 4.0 eV are highly desirable for air-stable electron-transporting semiconductors for facilitated charge injection.^37^ Ionization potential values determined by electron photoemission method in air (Figure S4, Table 2) were found to be comparable.

Theoretically determined highest occupied molecular orbital (HOMO α) values are also in agreement with the experimental results (Figure 1a and b). For the carbazole-containing radicals **1**–**3**, the HOMO α are delocalized on the donor fragment and the bridging carbazole moiety. The HOMO α of the dimethylacridan-containing radicals **4**–**6** are localized only on the donor fragment.

As for the singly occupied molecular orbitals (SOMO α), they are systematically localized on the electron accepting trivalent radical carbon containing the unpaired electron. The lowest unoccupied molecular orbitals (LUMO β) of all 6 compounds has the same localization as their SOMO α orbitals. Such electronic structures of the radicals **1**–**6** with large orbital separation and minimal overlap between the moieties will be found to heavily influence their absorption and emission properties.

### Photophysical Properties

**Figure 2:**
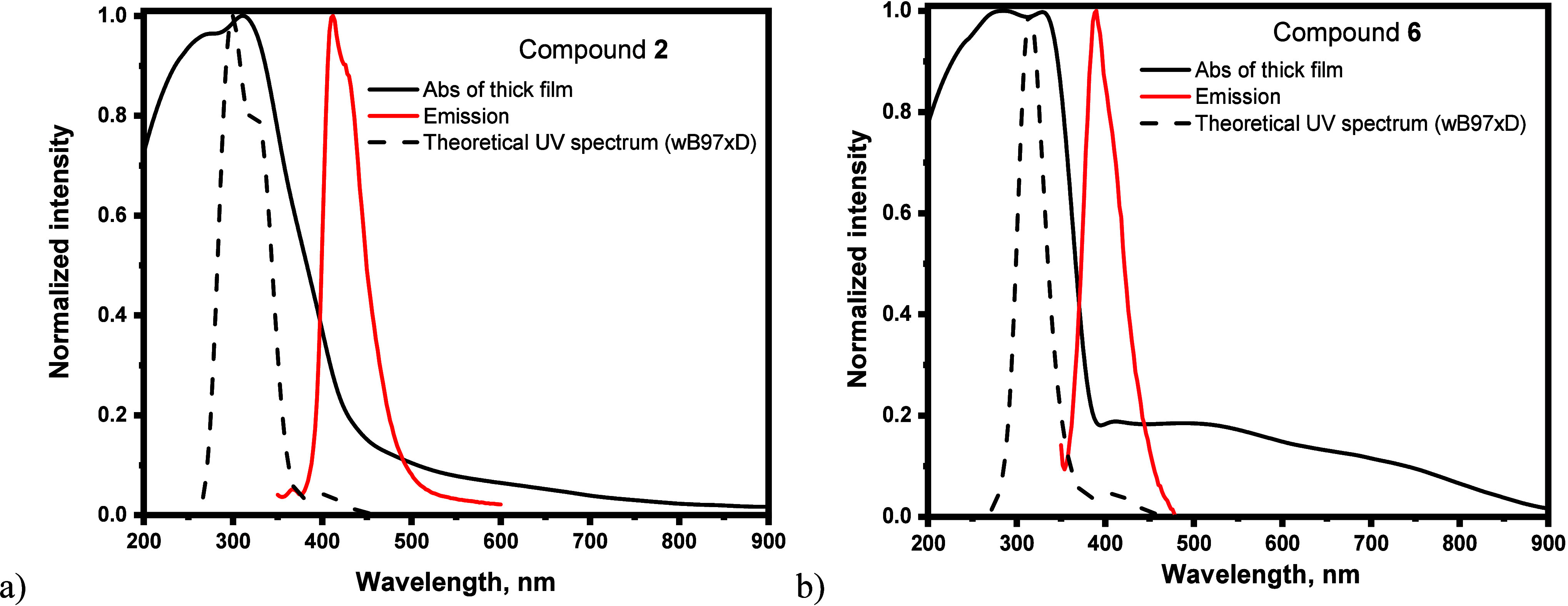
Normalized experimental UV–vis absorption and emission spectra of thick films of carbazole-based radical **2** (a) and dimethylacridan-carbazole-based radical **6** (b). The normalized theoretical spectra are also shown (dotted marks). Level of calculation here.

The absorption and emission spectra of toluene solutions and of the films of compounds **1**–**6** are shown in Figure 2 and Figure S5a–f, respectively, whereas the peak wavelengths of absorption (λ_abs_) and emission (λ_PL_) spectra are collected in Table 3.

**Table 3:** Photophysical Characteristics of Thin Films and Toluene Solutions of Stable Radicals **1**–**6**

Compound	**1**	**2**	**3**	**4**	**5**	**6**
λ_abs_, nm	291, 333, 402	291, 335, 439	289, 304, 338, 406	278, 293, 354	289, 313, 371	278, 334
λ_PL_, nm	365, 380^a^	412, 429^a^	380, 397^a^	408, 427^a^	413, 431^a^	408
PLQY of thin films	<1	<1	<1	<1	<1	<1
PLQY of aerated toluene solution, %	17.4	20.26	12.4	8.25	6.8	3
PLQY of deoxygenated toluene solution, %	27.8	30.76	18.46	14.6	11.5	7.25
CIE 1931, (x, y)	(0.16, 0.06)	(0.18, 0.05)	(0.16, 0.02)	(0.16, 0.04)	(0.16, 0.04)	(0.16, 0.04)

a 0–1 vibrational transitions.

The absorption spectra of toluene solutions of compounds **1**–**6** exhibit intense wide low-energy bands with onset wavelengths roughly around 360 nm. While similar observations hold true for the absorption spectra of the thin films of radicals, in the case of thick films, additional weak low-energy band tails can be easily observed at wavelengths longer than 360 nm (Figure 2a). Interestingly, these band tails in thick films extend up to the infrared region (IR), suggesting the narrow energy gaps. Nevertheless, the presence of these near IR absorption signals in the case of thick films suggest either emission from almost dark low-energy states or formation of excimer species in small percentage. This last conclusion is supported by the reasonable similarity between the absorption spectra of compounds **2** and **6** in THF solution and in thick films (Figure S5i, j). Indeed, only standard band enlargements can be observed for the spectra in thick films as compared to those in solution, indicating absence of visible intermolecular optical features. The TD–DFT (wB97XD/6-31+G**) calculations allow obtaining deep insights on the nature of the experimental absorption bands. The theoretical UV spectra of compounds **2** and **6** are very similar to the experimental absorption bands in toluene and thin films (Figure 2), exhibiting a weak low-energy band around 400 nm and a strong band located between 300 and 350 nm. These bands are characterized by transitions toward several excited states, but are dominated by doublet states (D_n_) D_0_ → D_8_, D_9_ transitions for radical **2** and by D_0_ → D_12_, D_14_ ones for the radical **6**. These transitions are a mixture of local-trityl excitations and trityl → central Cz charge-transfer excitations (Figure S8). The lowest-energy excited state D_1_ corresponds to the α SOMO → LUMO transition with considerably lower oscillator strengths of 0.0061 and 0.013 for **2** and **6**, respectively, as compared to 0.189 for D_0_ → D_8_ of compound **2** and 0.151 for D_0_ → D_12_ of compound **6**. These theoretical results suggest that the lowest energy transitions D_0_ → D_1_ do not contribute significantly to the absorption profiles of **2** and **6**.

Finally, the absorption spectrum calculated for one dimer of compound **2** taken as an example (Dimer 1) is shown in Figure S5k along with the theoretical absorption spectrum of the isolated compound **2**. The two spectra are very similar, exhibiting, for instance, the lowest-energy strong absorption around 330–340 nm.

Both experimental and theoretical results thus indicate that the optical properties of these radical compounds are determined by the intramolecular transitions in solution and in solid films.

The photoluminescence spectra of the toluene solutions of **1**–**6** are shown in Figure 2 and Figure S5. The main numerical results are collected in Table 3. As expected, the photoluminescence quantum yields (PLQY) of toluene solutions of **1**–**6** are sensitive to the presence of oxygen and are found to increase after deoxygenation. This is due to the quenching of the doublet emission by triplet oxygen, which is already described as originating from two possible mechanisms, either electron exchange in collision complexes,^38^ or quenching related to Dexter-type energy-transfer.^39^ The larger PLQY values of toluene solutions of carbazole-containing radicals (**1**–**3**) compared to those of dimethylacridan-based ones (**4**–**6**) may stem from intra- and/or intermolecular dynamic quenching in the excited state^40^ of the later compounds. It has been reported previously that the stable radical compounds tend to be nonemissive in the solid state due to dynamic quenching.^41,42^

The dilute toluene solutions of radicals **1**–**6** emit in the violet–blue region of the spectrum (Figure 2 and Figure S5), corresponding to CIE 1931 color coordinates which are close to the blue color standard of (0.14, 0.08) of the National Television System Committee (NTSC)^43^ (Table 3). The emission band is narrow, with fullwidth half-maximum (fwhm) values of 46 nm, suggesting pure color emission.

The emission of thin solid films of radicals **1**–**6** also occurs in the violet–blue region (Figure S5). While small differences between the PL wavelengths across the series of compounds **1**–**6** can be observed, one important observation is that the emission profile exhibits clear or traces of vibrational progression for all compounds, as indicated by the typical energy separation of 0.12–0.14 eV between the 0–0 and 0–1 vibrational peaks. These observations hold true for the emission spectra of compounds **1**–**6** in the cases of both toluene solutions and solid films. In view of the similar shape and energy of the emission spectra, it is tempting to correlate this very similar narrow and vibrationally structured blue emissions with the common presence in all compounds of the trityl fragment or with the central carbazole moiety. The very close resemblance between the CIE 1931 color coordinates across the series (Table 3) supports this hypothesis. While the trityl radical emission should be less prone to vibrational progression, it is interesting that none of the theoretical excited states are entirely localized on the carbazole moiety, thus leaving an ambiguity on the emission originating from local carbazole or from local-trityl excited states.

Nevertheless, the blue color emission of the radical compounds **1**–**6** peak at 341 and 356 nm, which is at odds with the red color emission reported for other radicals stemming from the relaxation of the lowest excited doublet state (D_1_)^5^ to the doublet ground-state (D_0_). Considering that the edges of absorption spectra of radicals **1**–**6** in the thick films are in the infrared region (Figure 2 and Figure S5), their lowest excited doublet states should also emit in the infrared region, which is not the case. Therefore, we are led to the conclusion that these compounds emit from highly lying excited states (D_n_)^42^ instead of emitting from their lowest excited state D_1_, thus violating the Kasha rule.

The TD–DFT calculations provide helpful insights with regard to the above conclusion. First, we note that the D_1_ excited states of compounds **1**–**6** are of charge-transfer nature (Figure S8), which is at odds with the observed vibrational structure of their emission profiles. Additionally, the geometry optimization of the D_1_ state of compound **2** at the wB97XD/6-31+G** level with tuned w = 0.0069 results in energy stabilization from 2.46 eV (503 nm, vertical excitation) down to 2.01 eV (615 nm), suggesting red emission color. Both D_1_ properties suggest that the blue emission of the compounds **1**–**6** cannot stem from this state.

As for the highest excited levels, Figures S8 and S9 indicate that most of the excited states from D_1_ through D_7_ (or D_8_) exhibit practically zero oscillator strengths, ranging 0.0001–0.01, which is indicative of their near or totally dark-state nature. The relatively high excited states (D_8_, D_9_ and D_12_, D_14_) of radicals **2** and **6**, respectively, afforded high oscillator strength values (larger than 0.15–0.18) and exhibit local-trityl transition nature. The D_3_ and D_2_ states of compounds **2** and **6** also exhibit similar local-trityl nature, yet their oscillators strengths are much lower (0.017 and 0.015, respectively). However, the internal conversion (IC) from the D_8_ or D_9_ down to D_3_ (D_2_) or to D_1_ seems to be hindered by different effects: (i) The energy gaps between D_4_ and D_5_, and D_6_ and D_7_ are higher than 0.22 eV (Figure S9a), which is a situation similar to the known case of triaryl methyl radical derivatives^42^ exhibiting energy gap between D2 and D1 higher than 0.46 eV. (ii) The MOs contributing to the excited states D_7_, D_6_, and D_5_ of compound **2** are localized on the bridging carbazole moiety toward the side carbazole moieties (Figures S9b and S10), which is different as compared to those contributing to the emissive states (D_8_ and D_9_) localized on the trityl radical fragment. One can suspect here a limited overlap between the vibrational wave function of D8 with those of the next low-lying excited states D_7_–D_5_. This last effect, along with the considerable energy splitting between D_7_ and the lower states, may result in slow internal conversion and favorable anti-Kasha emission. To further support these arguments, we have tried to record the emission of the toluene solutions and of the thin films of radicals **1**–**6** by exciting them at higher wavelength (550 nm), which energy of 2.25 eV is well above the energy of D_1_ (2.01 eV at the theoretical level), but below the one of the emission onsets (2.89 and 3.04 eV for **2** and **6**, respectively). However, these tests resulted in no emission, thus supporting the above analysis. It is worth highlighting at this point that compounds **1**–**6** demonstrate the most blue-shifted anti-Kasha doublet emission so far published in literature.

The short emission lifetime (Figure S6) of radicals **1**–**6** suggests that radiative decay occurs from the fluorescence that can boost device efficiency when used in the active layers of optoelectronic devices. To prove this prediction, the photostability test for compounds **1**–**6** was carried out by the similar procedure as it was previously reported for doublet emitters.^6^ Despite of the selection of rather harsh UV conditions (UV irradiation intensity of 1.1 mW/cm^2^ at the excitation wavelength of 365 nm), the emission intensity (I) of the toluene solution of compound **1** minimally changed during the irradiation time of 20 min in comparison to the emission intensity recorded before irradiation (I_0_) (Figure S7a and b). The rise of emission intensities of toluene solutions of compounds **4** and **6** was observed due to their emission sensitivity to active oxygen which is deactivated under UV excitation (Figure S7b, Table 3). Thus, relatively stable blue doublet emitters can be obtained using the stable radical acceptor and appropriate (stable and UV emitting) donor units.

### Charge-Transporting Properties

**Figure 3:**
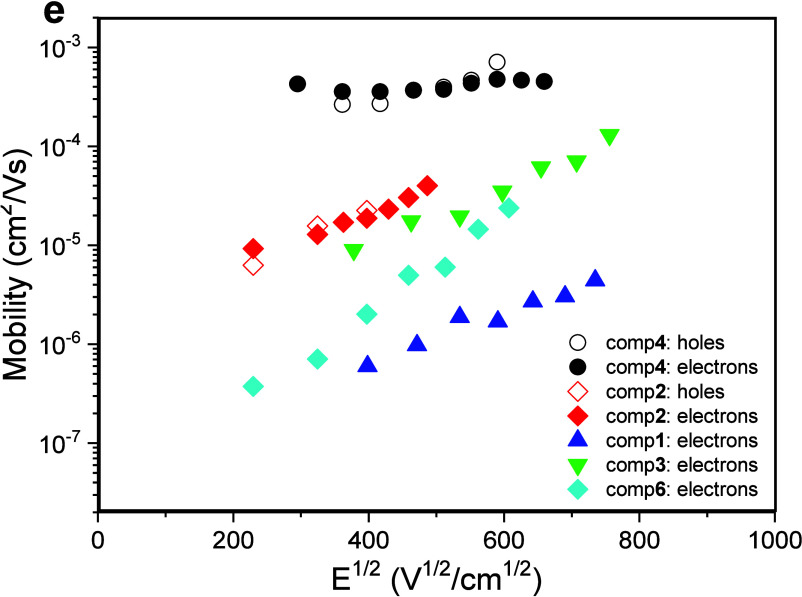
Electric field dependencies of charge carrier mobilities of radicals **1**–**6**.

Charge-transporting properties of the layers of stable radicals **1**–**6** were studied by time-of-flight (TOF) (Figure 3). Bipolar charge transport was observed for vacuum-deposited films of stable radicals **1**–**4** and **6**. The highest and most balanced electron and hole mobilities were observed for the dimethylacridan-based radical **4**. At an electric field of 3.5 × 10^5^ V/cm, hole and electron drift mobilities were found to be of 7.35 × 10^–4^ and of 4.51 × 10^–4^ cm^2^/V·s, respectively. Lower electron mobility values (2.29 × 10^–5^ to 2.33 × 10^–4^ cm^2^/V·s) were observed for compounds **1**, **3** and **6**. Bipolar and well-balanced charge transport was also observed for carbazole-based radical **2**.

To gain more insight into the relationship between the charge transporting properties and the chemical structures of stable radicals, two parameters entering Marcus’ equation for charge transfer^44^ were estimated at the DFT level: (*i*) the intramolecular reorganization energies for the isolated radicals **1**–**6** (λ, Table S2) for holes and electrons in vacuum. This parameter describes the influence of nuclei motion to the transporting electron.^45^ (*ii*) The electronic couplings (transfer integrals) between appropriate molecular orbitals of adjacent molecules: SOMOα - SOMOα for the hole transfer, and LUMO β - LUMO β for the electron transfer. For the two of the compounds, compound **1** (the worst performing compound) and compound **4** (the best performing one), selected dimers were constructed, and their geometries were fully optimized (see Figure S16). The dimers were constructed by positioning the central carbazole moiety one onto another in the same direction horizontally (dimer-1),in the opposite direction with the radical facing the side carbazole moiety (dimer-2), mirroring vertically (dimer-3), and mirroring in the opposite direction (dimer-4).

Additionally, the interaction energies for each dimer were also estimated, motivated by the assumption that stronger interaction energies should correspond to smaller contributions from the geometrical disorder in the films of the compounds.^46^

The results of these calculations are given in Figure S16 and in Table S2. The intramolecular reorganization energies for holes λ+ and electrons λ- were found to be only slightly lower for radicals **2** and **4**, nevertheless seeming to follow the trend of the experimental results. However, the dimers of radical **1** exhibit higher electronic coupling as compared to compound **4** for both SOMO α - SOMOα (average of 11.3 and 3.8 meV, respectively) and LUMO β - LUMO β (average of 29.3 and 18 meV, respectively), see Table S2.

Based on these results, we are led to the conclusion that the charge transport in these radicals is disorder dominated. Indeed, the comparison between the intermolecular interaction energies calculated from the dimers of these compounds indicates stronger interactions in the case of compound **4** as compared to compound **1** (28.7 and 27 kcal/mol, respectively, Table S4), seemingly leading to smaller disorder.^46^ This observation is in good agreement with the experimental results, as smaller disorder of radical **4** leads to better overall charge transporting properties.

The efficient bipolar hole and electron transport observed for compounds **1**–**6** can be attributed to their radical character of trityl carbon.^12^ The hole- and electron-transport is implemented through SOMO α (hole transfer) and LUMO β (electron transfer), both orbitals exhibiting identical space extension (on the trityl fragment) and shape, as opposed to the different properties of HOMO and LUMO in closed-shell compounds.^11^ Thus, by ignoring the spin of the electron for simplicity, both oxidation and reduction processes in the radical series **1**–**6** can be attributed to the capability of SOMO, localized on the trityl carbon atom, to lose or gain a single electron, which is at the origin of the bipolar character of the charge transfer in these radical compounds. Last but not least, the measured hole and electron mobility values of radical **4** are among the highest ones reported for stable radicals and comparable with those of the widely used tris(2,4,6-trichlorophenyl)methyl (TTM) series of radicals.^10^

### Photovoltaic Performance of PSCs with and without Radicals

**Figure 4:**
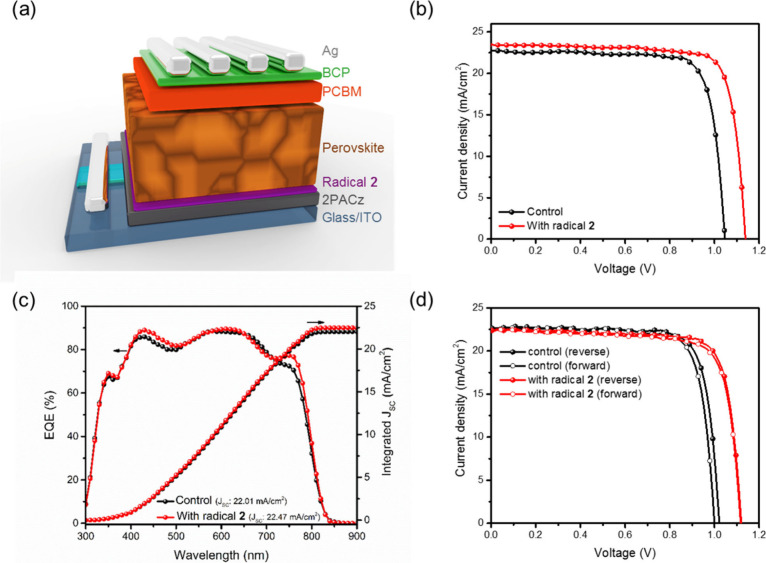
(a) Device architecture. (b) J–V curves from the best PCE results. (c) EQE characteristics. (d) Hysteresis of devices with and without radical **2**.

We use carbazole-based radicals **2** or **4** for the formation of the interfacial layer for hole extraction in inverted PSCs with the following architecture: ITO/2PACz/radical/perovskite/PEAI/PCBM/BCP/Ag (Figure 4(a)). This choice is based on its well-balanced charge transport and its compatibility with the perovskite layer, particularly with a well-matched HOMO energy level. Radicals **2** or **4** dissolved in chlorobenzene at a concentration of 0.5 mg/mL were spin-coated on top of the control HSL (2PACz). The current density–voltage (J–V) curves of the best devices under 100 mW cm^–2^ simulated AM 1.5 G irradiation are shown in Figure 4(b). The relevant device parameters are summarized in Table 4. The average PCE of the control device (without interfacial treatment by radicals) is of 18.49 ± 0.64%, with a short circuit current (*J*_SC_) of 22.12 ± 0.72 mA cm^–2^, an open circuit voltage (*V*_OC_) of 1.05 ± 0.01 V, and a fill factor (FF) of 79.46 ± 0.56%. Notably, the performance of the devices fabricated with HSL modified with radical **2** shows a significantly improvement, displaying a PCE of 20.02 ± 0.66%, with a *J*_SC_ of 22.37 ± 0.57 mA cm^–2^, and a *V*_OC_ of 1.13 ± 0.01 V and a FF of 79.09 ± 0.99%. We observed an increase in PCE of ca. 12% for the best radical **2** containing device, in comparison with that of the control device, which could be attributed to the increase in their *V*_OC_ and *J*_SC_. A comparable optimization approach was utilized to evaluate the influence of radical **4**, as depicted in Table S5, Figure S11 (Supporting Information) and Table 4. Despite radical **4** demonstrating the highest hole and electron mobility, its HOMO is not well-suited for fabricating Cs_0.18_FA_0.82_PbI_3_ PSCs. Consequently, the PCE values of 2PACz/radical **4**-based devices are lower compared to those 2PACz/radical **2**-based devices. Therefore, we focused on devices incorporated with radical **2** in the following studies. The corresponding external quantum efficiency (EQE) data of the devices with and without radical **2**, along with their relevant integrated *J*_SC_ are summarized in Figure 4(c). These devices exhibit good intensity in the range of 300 to 800 nm consistent with the J–V measurements. At the same time, the radical **2** containing device shows increased EQE values. The hysteresis index [HI = (PCE_reverse_ – PCE_forward_)/ PCE_reverse_] is used to evaluate the hysteresis degree of J–V measurements. As illustrated in Figure 4(d) and detailed in Table S6, the HI value is notably reduced in the device incorporating radical **2** (HI = 1.14%), compared with the control device (HI = 2.60%). A lower HI suggests that the carrier transport within the device is more efficient.

**Table 4:** Photovoltaic Parameters of Devices with and without Radical **2**^a^

Device	*J*_SC_ (mA/cm^2^)	*V*_OC_ (V)	FF (%)	PCE (%)
Control	22.12 ± 0.72 (22.78)	1.05 ± 0.01 (1.04)	79.46 ± 0.56 (80.0)	18.49 ± 0.64 (19.00)
With radical **2**	22.37 ± 0.57 (23.40)	1.13 ± 0.01 (1.14)	79.09 ± 0.99 (80.0)	20.02 ± 0.66 (21.29)

a The parameters of the best devices are listed in parentheses.

### Origins of the Improved Performance

Considering that surface properties can profoundly influence perovskite growth, we conducted contact angle measurements using water and diiodomethane (DIM) as probe liquids, as shown in Figure S12 and summarized in Table S7. The average water contact angles of the HSL (2PACz) and HSL/interlayer (2PACz/radical **2**) are 43.36° and 57.58°, respectively, indicating an increase in hydrophobicity due to the addition of radical **2**. Additionally, the average DIM contact angles are 17.66° and 20.18° for 2PACz and 2PACz/radical **2**, respectively. The surface tension of polar (γ_polar_) and dispersive (γ_dispersive_) components as well as total surface energy (γ_total_) can be calculated from the water and DIM contact angles using the Wu model.^47^ As listed in Table S7, the calculated γ_total_ values of the surface of 2PACz and 2PACz/radical **2** are 72.63 and 65.20 mN m^–1^, respectively. This indicates that radical **2** can significantly reduce the barrier of high surface energy, thereby greatly affecting the formation of perovskite.^48^

The energy levels of valence band (E_VB_) for HSLs were estimated by ultraviolet photoelectron spectroscopy (UPS) according to the formula E_VB_ = 21.22 – (E_cutoff_ – E_onset_) and the E_VB_ of perovskite used in this study was obtained from literature.^49^ As shown in Figure S13a and b, the VB level of HSL was reduced from −5.84 to −5.42 eV for 2PACz and 2PACz/radical **2**, respectively. The upshift of 420 meV of the VB level indicates the modification of the surface chemical state by radical **2** and provides an additional driving force for hole extraction,^50^ resulting in an improved *V*_OC_.

To understand how radicals **2** and **4** affect the electronic properties of the HSLs, photoelectron spectroscopy in air (PESA) and Kelvin probe (KP) measurements were performed to determine the work function. As shown in Figures S13c and d, the incorporation of radicals **2** and **4** resulted in reductions in the IP/WF levels to −5.35/–5.29 eV and −5.37/–5.31 eV, respectively, compared to the values of −5.62 and −5.36 eV for 2PACz. This decrease in IP and WF suggests that radicals **2** and **4** effectively modify the HSLs’ electronic structure, potentially leading to improved performance in PSCs by promoting better hole transport and reducing recombination losses.

We conducted the analysis of perovskite crystal morphology by scanning electron microscopy (SEM), as shown in Figure S14. We observed similar surface morphology for both the control and the radical **2** treated perovskite. However, it is noteworthy that the average grain size of the radical **2** treated perovskite is slightly larger than that of the control sample. This increase in perovskite grain size, along with fewer grain boundaries, can be attributed to the effective reduction of defect densities. Such a reduction in defects can diminish trap-assisted recombination in PSCs.

To further examine the difference of perovskite growth on 2PACz and 2PACz/radical **2**, we conduct X-ray diffraction (XRD) measurements, as depicted in Figure S15(a). Both perovskite films display the characteristic diffraction peaks at 13.8°, 19.7°, 24.2°, and 28.1° corresponding to the (1 0 0), (1 1 0), (1 1 1) and (2 0 0) planes of the cubic Cs_0.18_FA_0.82_PbI_3_ phase. For the more detailed analysis, we investigate the full width at half-maximum (fwhm) of (1 0 0) diffraction peak. As illustrated in Figure S15(b), a slightly narrower fwhm can be observed for the normalized signal of perovskite film grown on radical **2**, implying the formation of better crystal orientations. The higher quality of perovskite is derived from the suitable surface energy, leading the mitigation of the interfacial defects,^24^ which is in line with the SEM results.

To understand the causes of improved performance of 2PACz/radical **2**-based PSCs, the charge carrier dynamics in the perovskites were analyzed. Figure S15(c) shows the steady-state photoluminescence (PL) spectra of perovskite coated on the different samples. The 2PACz/radical **2**/perovskite sample showed a lower PL intensity than 2PACz/perovskite sample, which suggests that the photoinduced holes can be efficiently transported from the perovskites to HSL with the introduction of radical **2**. To further verify such a conclusion, the time-resolved PL (TRPL) measurement was carried out (Figure S15(d)). The biexponential decay fitting parameters are listed in Table S8. It reveals that the average PL lifetime of 2PACz/radical **2**/perovskite sample of 243.5 is higher than that of 2PACz/perovskite sample (200.6 ns). The reductions in PL intensity and TRPL lifetime give evidence that radical **2** can effectively suppress charge recombination and improve interfacial charge transfer by passivating interfacial defects, which can be attributed to the increase in *V*_OC_ of the device incorporating radical **2**.^24−26,51^

**Figure 5:**
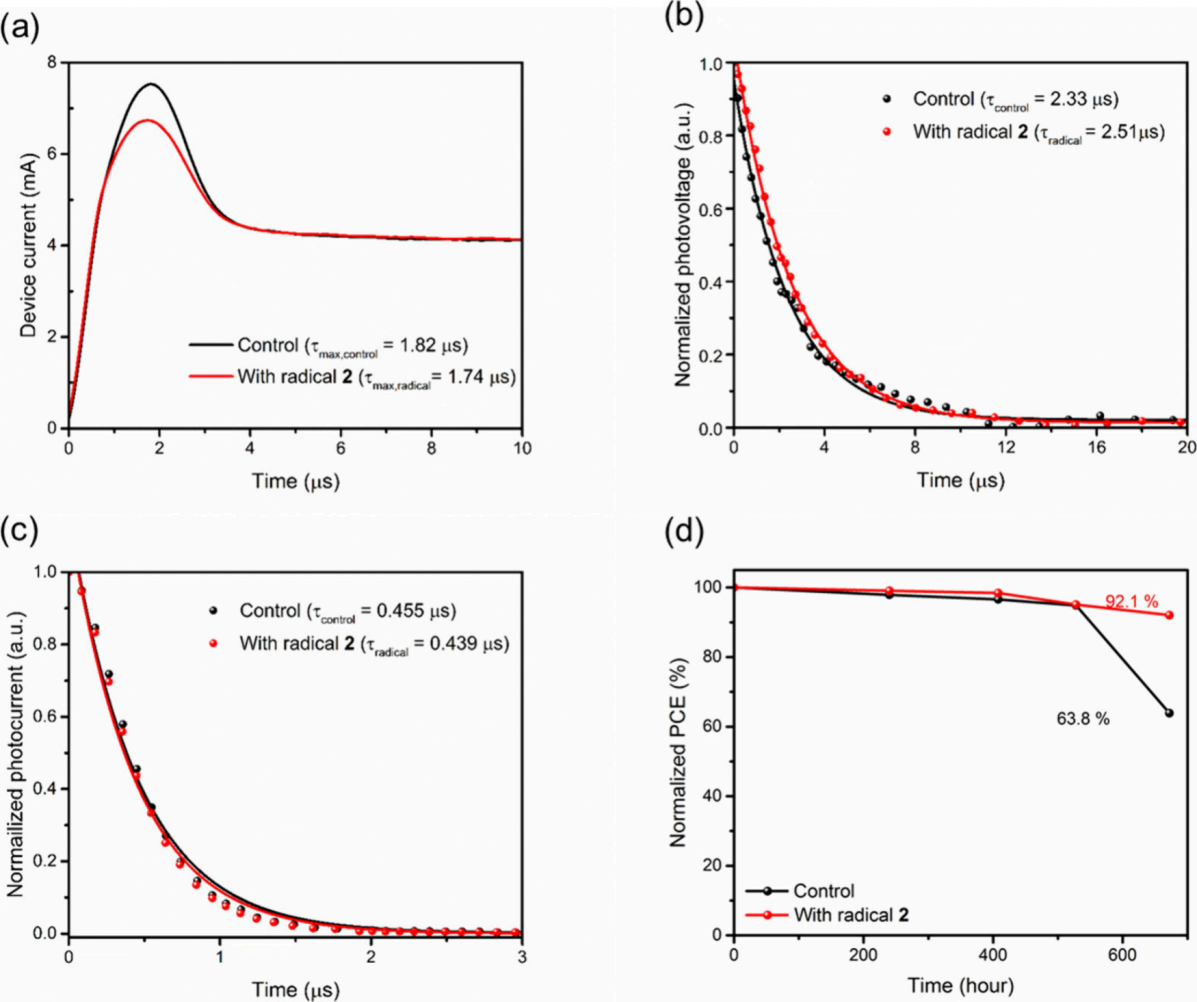
(a) Photo-CELIV, (b) TPV, and (c) TPC curves of the devices with and without radical **2**. (d) Long-term stability of the unencapsulated devices tested in argon-filled glovebox.

To shed light on the effect of radical interlayer on charge carriers transport and recombination in the devices, the charge extraction of photogenerated charge carriers by linearly increasing voltage (photo-CELIV), transient photovoltage (TPV), and transient photocurrent (TPC) measurements were performed. From the curves shown in Figure 5(a), a slightly shorter τ_max_ is obtained for devices incorporating radical **2**, giving the higher CELIV mobility of 2.74 × 10^–3^ cm^2^ V^–1^ s^–1^ for the device based on 2PACz/radical **2**, compared to that of 1.90 × 10^–3^ cm^2^ V^–1^ s^–1^ observed for the control device. Meanwhile, the charge carrier lifetimes can be measured by TPV under open-circuit conditions, as shown in Figure 5(b). The 2PACz/radical **2**-based device exhibits a much slower TPV decay profile than the control device, indicating the carrier lifetime in PSC with the incorporation of radical **2** is longer than that in control device. The longer lifetime implies the suppression of charge recombination within the device. TPC was conducted under short-circuit conditions to evaluate the charge transfer in the devices, as shown in Figure 4(c). The 2PACz/radical **2**-based device shows a much faster TPC profile, indicating better charge extraction at the HSL interface, which is in line with increased mobility and appropriate energy level alignment within the device.

To gain further insight into the stability of PSCs, we executed the long-term stability of the control and the optimized devices without encapsulation in argon-filled glovebox. As shown in Figure 4(d), after more than 600 h of storage, PCE of the control PSC rapidly decreased to 63.8% of its original value. In contrast, the device incorporating with radical **2** maintained 92.1% of its initial PCE after the same duration. This improved stability can be attributed to defect passivation and the presence of a perovskite film with better crystal orientation.

## Conclusion

Bipolar electroactive stable radicals containing the trityl radical carbon moiety were synthesized via the Friedel–Crafts alkylation reaction. The design strategy of trityl carbon radical protection by methyl groups was successfully implemented. The radicals were found to be thermally and electrochemically stable. The radicals with methyl substituted phenyl groups show superior photochemical stability. According to the experimental results as well as the results of TD-DFT calculations, the blue emission of radicals violates the Kasha rule and originates from higher excited states. The radicals demonstrate the most blue-shifted anti-Kasha doublet emission reported so far with high color purity full width at half-maximum of 46 nm and relatively high photoluminescence quantum yields of deoxygenated toluene solutions reaching 31%. The stable radicals demonstrate extremely equilibrated bipolar charge transport with charge mobility values reaching 10^–4^ cm^2^/V·s at strong electric fields. The perovskite solar cells containing a layer of stable radicals as a proper interlayer show encouraging power conversion efficiency of 21% and robust stability. Overall, this work provides an efficient design strategy of multifunctional electroactive stable radicals that can expand the application possibilities not only in OLEDs but also in solar cells.

## Experimental Section

### Instrumentation

^1^H NMR spectra were recorded using a Bruker Avance III apparatus (400 MHz). The samples were prepared by dissolving around 20 mg of the material in 1 mL of deuterated chloroform (CDCl_3_). Hydrogen nuclei ^1^H were excited by using the frequency of 400 MHz. The data are presented as chemical shifts (δ) in ppm (in parentheses: multiplicity, integration, coupling constant).

IR spectra were recorded by a Vertex 70 Bruker spectrometer equipped with an ATR attachment with a diamond crystal over frequencies of 600–3500 cm^–1^ with a resolution of 5 cm^–1^ over 32 scans.

The electron paramagnetic resonance (EPR) was performed by employing a Bruker Elexsys 560 EPR spectroscopy system.

UV–vis spectra of the films of radicals **1**–**6** were recorded on a spectrometer Avantes AvaSpec-2048XL. Photoluminescence (PL) spectra were recorded on a FLS980 fluorescence spectrometer.

Differential scanning calorimetry (DSC) measurements were carried out using a TA Instruments Q2000 thermosystem. The samples were examined at a heating/cooling rate of 10 °C/min under nitrogen atmosphere.

Thermogravimetric analysis (TGA) was performed on a TA Instruments Q50 analyzer. The heating rate was 20 °C/min. The measurements were performed under nitrogen atmosphere.

Cyclic voltammetry measurements were performed using a glassy carbon working electrode (a disk with the diameter of 2 mm) in a three-electrode cell with an Autolab Type potentiostat–galvanostat. The measurements were carried out for the solutions in dry dichloromethane containing 0.1 M tetrabutylammonium hexafluorophosphate at 25 °C. The scan rate was 50 mV/s, and the sample concentration was 10^–3^ M. The potentials were measured against silver as a reference electrode. Platinum wire was used as a counter electrode. The potentials were calibrated with the standard ferrocene/ferrocenium (Fc/Fc^+^) redox system.^52^

The absorption properties were examined through time-dependent density functional theory (TD-FT) calculations to characterize the excited states of the target compounds. Geometry optimization at the ground state was performed at the B3LYP/6-31+G** level of theory. The excited states were calculated at the ωB97XD19/6-31+G** level of theory with a tuned w value of 0.0069. The geometry of dimers was optimized at the wB97XD/6-31+G** level of theory.

In the setup for the TOF measurements, an EKSPLA NL300 laser (excitation wavelength of 355 nm), a 6517B electrometer (Keithley), and a TDS 3032C oscilloscope (Tektronix) are used. To test the ability to transport holes and electrons of compounds **1**–**6**, TOF transients under positive and negative polarity of applied electric fields are recorded. When two different slopes (intercepts of which give the transit times (t_tr_)) are obtained from the corresponding TOF transients build in log–log scales, hole and electron mobilities are calculated according to the equation μ = d^2^/(U × t_tr_) taking transit times t_tr_ from the photocurrent transients at applied voltage (U) and thicknesses of the layers (d) measured by the charge extraction by linearly increasing the voltage (CELIV) technique assuming a dielectric constant ε = 3 for the studied compounds. The samples for the TOF are fabricated on prepatterned and precleaned ITO glass substrates with sheet resistance of 15 Ω/sq. The films for the TOF measurements are vacuum deposited with Kurt J. Lesker equipment integrated into glovebox.

### Fabrication of PSC

The inverted perovskite solar cells were fabricated with the architecture of glass/ITO/2PACz/with or without radicals/Cs_0.18_FA_0.82_PbI_3_/PEAI/PCBM/BCP/Ag.

ITO substrates were precleaned in an ultrasonic bath, sequentially with abstergent aqueous solution, deionized water, acetone, and isopropyl alcohol, for 20 min each, then dried under a stream of N_2_. The substrates were further treated with plasma for 10 min before the deposition of the hole-selective layer (HSL). A self-assembled layer of 2PACz HSL was spin-cast (3000 rpm, 30 s) on ITO and then baked at 100 °C for 10 min. The 2PACz precursor solution was prepared by dissolving 2PACz in ethanol at a concentration of 1 mg/mL. For interfacial engineering between HSL and perovskite, the radical **2** dissolved in chlorobenzene was spin-cast (5000 rpm, 30 s) on the top of 2PACz. The prepared solution was stirred overnight and was put in an ultrasonic bath for 15 min before it was used. The perovskite precursor was prepared by dissolving FAI (172 mg), PbI2 (507 mg), and CsCl (30 mg) in a 1 mL mixture of DMF and DMSO (4:1, v/v). The perovskite layer was deposited on the substrate in a two-step manner: first at 1000 rpm for 10 s and then at 5000 rpm for 30 s. During the second step, chlorobenzene (0.15 mL) was dropped on the spinning substrate; the sample was then annealed at 150 °C for 30 min. For the PEAI surface passivation, PEAI dissolved in IPA at a concentration of 1.5 mg/mL was spin-coated (5000 rpm, 30 s) on the top of the perovskite, followed by annealing at 100 °C for 1 min. For the electron transport layer, a solution of PC_61_BM in chlorobenzene (20 mg/mL) was spin-coated (2000 rpm, 30 s) on the top of a PEAI layer. For the hole blocking layer, a solution of BCP in IPA (0.5 mg/mL) was spin-coated (6000 rpm, 10 s) onto the PC_61_BM layer. Finally, the devices were completed by evaporating Ag (100 nm) in a vacuum chamber; the active area of this electrode was fixed at 10 mm^2^.

Device performance data were collected within a glovebox. The current–voltage (I–V) characteristics of the devices were measured employing a computer-controlled Keithley 2400 source measurement unit (SMU) and a Dyesol simulator (AAA Class Solar Simulators) under AM 1.5 illumination (100 mW/cm^2^). The light intensity was calibrated using a standard Si reference cell and a KG-5 filter. EQE spectra were recorded using an Enlitech QE-R spectral response measurement system to standardize the current densities of the devices. PL spectra were acquired using an Edinburgh FLS1000 photoluminescence spectrometer, employing an excitation wavelength of 550 nm. TRPL spectra were also recorded using an Edinburgh FLS1000 photoluminescence spectrometer with a pulse laser featuring a wavelength of 447 nm. The laser was operated with a 500 ns excitation duration. The comprehensive characterization platform Paios (Fluxim AG) was employed to assess the optoelectronic properties of PSCs, including photo-CELIV, TPC, and TPV measurements. Typically, photo-CELIV determines the mobility of the faster carrier components, and mobility (μ) can be calculated from the equation , where d is the thickness of the active layer, A is the ramp rate of the applied voltage pulse (using 100 V ms^–1^ in this work), and τ_max_ is the time when the current density reaches the maximum value. The factor (1 + 0.36·Δ*J/J*_(0)_) is an empirical correction for the redistribution of the electric field, where *J*_(0)_ is the displacement current offset, and Δ*J* is the current overshoot.

Crystallinity information was obtained using a Malvern Panalytical Empyrean X-ray diffractometer with Cu Kα radiation (λ = 0.1542 nm) and a step size of 0.02°. The static contact angles measurements were recorded using the First ten angstroms/FTA-1000B apparatus. SEM images were recorded on a HITACHI S-5200 scanning electron microscope. PESA and KP were collected using Riken AC-2 photoelectron spectrometer and KP Technology, respectively. KP results were averaged from 300 counts and calculated from the contact potential difference (CPD) relative to a gold reference with a work function of −4.78 eV.

### Materials

9*H*-Carbazole, N-bromosuccinimide, iodoethane, sodium hydroxide, benzophenone, sodium borohydride, *tert*-butyl chloride, aluminum chloride, copper, copper(I)chloride, 1,10-phenanthroline, potassium carbonate, boron trifluoride diethyl etherate, cesium carbonate, tetrakis(triphenylphosphine)palladium(0), sodium *tert*-butoxide, tris(dibenzylideneacetone)dipalladium(0), 2-dicyclohexylphosphino-2′,4′,6′-tri-isopropylbiphenyl (Xphos), 2,6-dimethylbenzoyl chloride, 2,6-dimethylphenylboronic acid, and potassium *tert*-butoxide were purchased either from Sigma-Aldrich or Fluorochem. [2-(9H-Carbazol-9-yl)ethyl]phosphonic acid (2PACz, >98%, TCI), cesium chloride (CsCl, 99.9%, ultra dry, Alfa Aesar), formamidinium iodide (FAI, >99.99%, Greatcell Solar), lead iodide (PbI_2_, 99.9985%, Alfa Aesar), ethanol (anhydrous, ECHO), bathocuproine (BCP, Aldrich), dimethyl sulfoxide (DMSO, Sigma–Aldrich), N,N-dimethylformamide (DMF, Arcos Organics), phenethylammonium iodide (PEAI, Greatcell Solar), and PC_61_BM (Nano-C) were used as received. All the solvents were purchased commercially. Toluene, xylene, DMF, and THF were distilled and dried over molecular sieves before used.

#### 3-Bromo-9H-carbazole

This was synthesized according to the reported procedure.^53^

#### 3,6-Di-*tert*-butyl-9H-carbazole

This was synthesized according to the reported procedure.^54^

#### 3-Bromo-9-ethyl-9H-carbazole

This was synthesized according to the reported procedure.^55^

#### Bis(2,6-dimethylphenyl)methanone

This was synthesized according to the reported procedure.^56^

#### Diphenylmethanol

This was synthesized according to the reported procedure.^57^

#### Bis(2,4-dimethylphenyl)methanol

Here, 1g of bis(2,4-dimethylphenyl)methanone was dissolved in 20 mL of methanol and cooled down. Then sodium borohydride was added slowly by small portions under constant stirring until the bubbling stopped. The reaction mixture was poured into 100 mL of water. The product was filtrated and dried. Yield of white powder was 90%. MM = 240.35 g/mol. MS (*m*/*z*): found 239. ^1^H NMR (400 MHz, CDCl_3_) δ 7.15–6.99 (m, 2H), 7.15–6.98 (m, 2H), 6.98–6.78 (m, 4H), 6.93 (d, *J* = 20.5 Hz, 4H), 6.00 (d, *J* = 4.7 Hz, 1H), 6.00 (d, *J* = 4.7 Hz, 1H), 2.20 (d, *J* = 26.5 Hz, 12H), 2.20 (d, *J* = 26.5 Hz, 12H).

#### Bis(2,6-dimethylphenyl)methanol

Here, 1 g of bis(2,6-dimethylphenyl)methanone (**2**) was dissolved in 20 mL of methanol and cooled down. Then sodium borohydride was added slowly by small portions under constant stirring until the bubbling stopped. The reaction mixture was poured into 100 mL of water. The product was filtrated and dried. Yield of white powder was 90%. MM = 240.35 g/mol. MS (*m*/*z*): found 239. ^1^H NMR (400 MHz, CDCl_3_) δ 7.48 (d, *J* = 11.9 Hz, 2H), 7.37 (t, *J* = 6.5 Hz, 2H), 7.04 (t, *J* = 7.6 Hz, 1H), 6.87 (d, *J* = 7.5 Hz, 2H), 5.71 (s, 2H), 2.88–1.99 (m, 12H).

#### 3′,6′-Di-*tert*-butyl-9-ethyl-9*H*-3,9′-bicarbazole

Here, 1 g (1 eqv.) of 3-bromo-9-ethyl-9*H*-carbazole was added to a Shlenk flask containing 1.33 g (1.3 eqv.) of 3,6-di-*tert*-butyl-9*H*-carbazole, 0.02 g (0.01 eqv.) of copper powder, 0.03g (0.01 eqv.) of copper(I)chloride, 0.13g (0.2 eqv.) of 1,10-phenanthroline, 1 g (2 eqv.) of potassium carbonate, and 7 mL of xylene. The reaction mixture was heated at 135 °C for 24 h. After cooling down, the reaction mixture was filtrated through a 2 cm layer of Celite and washed with DCM. All volatiles were evaporated in vacuum. The product was recrystallized from methanol. Yield of white powder was 64%. MM = 472.68 g/mol. MS (*m*/*z*): found 486. ^1^H NMR (400 MHz, CDCl_3_) δ 8.13 (d, *J* = 16.6 Hz, 2H), 7.99 (d, *J* = 7.8 Hz, 1H), 7.51 (s, *J* = 14.1 Hz, 2H), 7.48–7.33 (m, 3H), 7.24 (d, *J* = 8.6 Hz, 1H), 7.20–7.16 (m, 2H), 4.40 (q, *J* = 7.1 Hz, 2H), 1.58–1.43 (m, 8H), 1.40 (d, *J* = 6.8 Hz, 13H).

#### Synthesis Procedure of Intermediate Compounds **I1**–**I3**

Here, 1 eqv. of 3′,6′-di-*tert*-butyl-9-ethyl-9H-3,9′-bicarbazole and 1 eqv. of corresponding diphenylmethanol were dissolved in dry DCM under argon atmosphere. The flask with the solution was placed into a vessel with ice and left for stirring until cooling down to 0–5 °C. Then 1. eqv of boron trifluoride diethyl etherate was added very slowly by drops. The solution was left for stirring for 24 h at room temperature. After completing the reaction, the stirring reaction mixture was quenched with sodium carbonate water solution until the color disappeared. The product was extracted from water using dichloromethane and dried over anhydrous sodium sulfate. All volatiles were evaporated in vacuum. The products were recrystallized from methanol.

##### 6-(Bis(2,4-dimethylphenyl)methyl)-3′,6′-di-*tert*-butyl-9-ethyl-9H-3,9′-bicarbazole (Precursor **I1**)

Yield of white powder was 80%. MM = 695.01 g/mol. MS (*m*/*z*): found 694. IR ν_max_ (ATR diamond): 3005 (−C–H Ar), 2963, 2958, 2954 (−C–H aliph); 1453, 1448, 1441, (−C–N−); 1296, 1270, 1261((CH_3_)_3_C−); 1392, 1384, 1380 (CH_3_−). ^1^H NMR (400 MHz, CDCl_3_) δ 8.13–7.85 (m, 2H), 7.50–7.08 (m, 15H), 7.07–6.70 (m, 5H), 6.69–6.19 (m, 7H), 4.32 (qt, *J* = 14.2, 7.1 Hz, 2H), 1.64–1.31 (m, 12H), 1.21 (d, *J* = 13.7 Hz, 10H).

##### 6-Benzhydryl-3′,6′-di-*tert*-butyl-9-ethyl-9H-3,9′-bicarbazole (Precursor **I2**)

Yield of white powder was 70%. MM = 638.90 g/mol. MS (*m*/*z*): found 680. IR ν_max_ (ATR diamond): 3024 (−C–H Ar); 2964, 2959, 2941 (−C–H aliph.); 1458, 1450, 1447 (−C–N−); 1362, 1350, 1346 ((CH_3_)_3_C−); 1380, 1376, 1372 (CH_3_−). ^1^H NMR (400 MHz, CDCl_3_) δ 8.02 (dd, *J* = 39.2, 13.5 Hz, 1H), 7.55–7.21 (m, 1H), 7.17–6.93 (m, 1H), 6.89–6.46 (m, *J* = 60.8, 29.5 Hz, 1H), 6.39–6.16 (m, 1H), 4.34 (d, *J* = 7.3 Hz, 1H), 1.72–1.01 (m, 6H), 0.81 (s, 1H).

##### 6-(Bis(2,6-dimethylphenyl)methyl)-3′,6′-di*tert*-butyl-9-ethyl-9H-3,9′-bicarbazole (Precursor **I3**)

Yield of white powder was 80%. MM = 695.01 g/mol. MS (*m*/*z*): found 694. IR ν_max_ (ATR diamond): 3028(−C–H Ar); 2959, 2956, 2948 (−C–H aliph.); 1495, 1489, 1483 (−C–N−); 1326, 1318, 1293((CH_3_)_3_C−); 1473, 1465, 1430 (CH_3_−). ^1^H NMR (400 MHz, CDCl_3_) δ 8.11 (t, *J* = 12.8 Hz, 3H), 7.99 (d, *J* = 7.8 Hz, 1H), 7.62–7.30 (m, 5H), 7.27–7.21 (m, 2H), 4.40 (q, *J* = 7.1 Hz, 2H), 1.43 (q, *J* = 7.3 Hz, 22H), 1.18 (s, 2H).

#### 2,7-Di-*tert*-butyl-10-(9-ethyl-9H-carbazol-3-yl)-9,9-dimethyl-9,10-dihydroacridine

Here, 1 g (1 eqv.) of 3-bromo-9-ethyl-9*H*-carbazole, 1.85 g (1.05 eqv.) of 2,7-di-*tert*-butyl-9,9-dimethyl-9,10-dihydroacridine, 0.78 g (1.5eqv.) of sodim *tert*-butoxide, 0.1 g (0.04 eqv.) of bis(tri-*tert*-butylphosphine)palladium(0), and 10 mL of toluene were loaded into a Shlenk flask. Air was removed with a vacuum pump, and the flask was filled with argon. The reaction mixture was left for stirring at 110 °C for 24 h. Then the product was filtrated through 2 cm layer of Celite, washed with DCM. All volatiles were under reduced pressure. The product was recrystallized from methanol. Yield of gray powder was 70%. MM = 514.76 g/mol. MS (*m*/*z*): found 514. ^1^H NMR (400 MHz, CDCl_3_) δ 8.17–7.92 (m, 2H), 7.68–7.33 (m, 6H), 7.32–7.15 (m, 3H), 6.97 (s, *J* = 33.6 Hz, 2H), 4.49 (q, *J* = 7.0 Hz, 2H), 1.82 (s, *J* = 14.6 Hz, 6H), 1.57 (t, *J* = 7.1 Hz, 3H), 1.34 (s, 18H).

#### Synthesis Procedure of Intermediate Compounds **I4**–**I6**

Here, 1 eqv. of 2,7-di-*tert*-butyl-10-(9-ethyl-9H-carbazol-3-yl)-9,9-dimethyl-9,10-dihydroacridine and 1 eqv. of corresponding diphenylmethanol were dissolved in dry DCM under argon atmosphere. The flask with the solution was placed into a vessel with ice and left for stirring until cooling down to 0–5 °C. Then 1 eqv. of boron trifluoride diethyl etherate was added very slowly by drops. The solution was left for stirring for 24 h at room temperature. After completing the reaction, the stirring reaction mixture was quenched with sodium carbonate water solution until the color disappeared. The product was extracted from water using DCM and dried over anhydrous sodium sulfate. All volatiles were evaporated in vacuum. The product was recrystallized from methanol.

##### 10-(6-(Bis(2,4-dimethylphenyl)methyl)-9-ethyl-9H-carbazol-3-yl)-2,7-di*tert*-butyl-9,9-dimethyl-9,10-dihydroacridine (Precursor **I4**)

Yield of white powder was 80%. MM= 737.09 g/mol. MS (*m*/*z*): found 260. ^1^H NMR (400 MHz, CDCl_3_) δ 8.04–7.72 (m, 1H), 7.39 (ddd, *J* = 51.2, 30.9, 8.6 Hz, 5H), 7.04–6.74 (m, 5H), 6.52 (ddd, *J* = 58.8, 48.9, 22.2 Hz, 4H), 4.31 (dd, *J* = 51.2, 44.6 Hz, 2H), 1.57–1.34 (m, 6H), 1.21 (s, *J* = 4.2 Hz, 30H), 0.94–0.63 (m, 5H). IR ν_max_ (ATR diamond): 3024 (−C–H Ar); 2956, 2951, 2938(−C–H aliph.); 1490, 1482, 1471 (−C–N−); 1296, 1288, 1283 ((CH_3_)_3_C−); 1362, 1353 (CH_3_−).

##### 10-(6-Benzhydryl-9-ethyl-9H-carbazol-3-yl)-2,7-di*tert*-butyl-9,9-dimethyl-9,10-dihydroacridine (Precursor **I5**)

Yield of white powder was 70%. MM= 680.41 g/mol. MS (*m*/*z*): found 681. ^1^H NMR (400 MHz, CDCl_3_) δ 7.87–7.65 (m, 1H), 7.61–7.21 (m, 5H), 7.13–6.48 (m, 13H), 6.27–5.95 (m, 2H), 4.40–4.00 (m, 2H), 1.82–1.31 (m, 9H), 1.30–0.97 (m, 15H), 0.77 (dd, *J* = 21.6, 13.4 Hz, 2H). IR ν_max_ (ATR diamond): 3004(−C–H Ar); 2955, 2950, 2941(−C–H aliph.); 1490, 1486,1458 (−C–N−); 1269, 1228 (CH_3_)_3_C−); 1324, 1312, 1308 (CH_3_−).

##### 10-(6-(Bis(2,6-dimethylphenyl)methyl)-9-ethyl-9H-carbazol-3-yl)-2,7-di*tert*-butyl-9,9-dimethyl-9,10-dihydroacridine (Precursor **I6**)

Yield of white powder was 72%. MM= 737.09 g/mol. MS (*m*/*z*): found 737. ^1^H NMR (400 MHz, CDCl_3_) δ 7.53–7.22 (m, 5H), 7.11–6.71 (m, 8H), 6.68–6.36 (m, 4H), 6.25–5.94 (m, 2H), 4.44–4.05 (m, *J* = 71.9, 36.0 Hz, 2H), 2.32–2.03 (m, 18H), 2.02–1.74 (m, 4H), 1.59–1.33 (m, 7H), 1.27–1.01 (m, 16H). IR ν_max_ (ATR diamond): 3028 (−C–H Ar); 2951, 2958, 2964 (−C–H aliph.); 1487 1458, 1454 (−C–N−); 1392, 1376, 1362 ((CH_3_)_3_C−); 1350, 1348 (CH_3_−).

#### Synthesis Description of Radicals **1**–**6**

Under an argon atmosphere and in the dark, the corresponding intermediate compounds **I1–I6** (1.00 eqv.) are dissolved in dry THF (40 mL) (Figure 1). Then KOtBu (2.00 eqv.) is added, and the solution becomes claret colored immediately. The solution is stirred for 1.5 h in the dark at room temperature, and then *p*-chloranil (2.7 eqv.) is added. The solution was stirred for further 1.5 h. When the reaction finished, its mixture is filtrated through a 2 cm layer of Celite and washed with ethyl acetate. All the volatiles are removed under vacuum, and the products are recrystallized from methanol. The EPR method is used to confirm the radical form (Figure S1). This technique includes the measurement of the energy absorption generated under microwave irradiation and varying the magnetic field. EPR spectroscopy results confirmed the existence of the unpaired electron in the molecular structure of all the target compounds with the g value variating from 2.00359 to 2.00546 (g = 2.0023 for free electron theoretically^58^).

##### 6-(Bis(2,4-dimethylphenyl)methyl)-3′,6′-di-*tert*-butyl-9-ethyl-9H-3,9′-bicarbazole Radical (Compound **1**)

Yield of pale-yellow powder is of 28% (0.063 g). IR ν_max_ (KBr tablet): 3007 (−C–H Ar); 2961, 2946, 2940 (−C–H aliph.); 1491, 1487, 1479 (−C–N−); 1388, 1385, 1374 ((CH3)3C−); 1326, 1316, 1309 (CH3−).

##### 6-Benzhydryl-3′,6′-di-*tert*-butyl-9-ethyl-9H-3,9′-bicarbazole Radical (Compound **2**)

Yield of dark brown powder is of 19% (0.096 g). IR ν_max_ (KBr tablet): 3020 (−C–H Ar), 2987, 2983 (−C–H aliph.); 1490, 1483, 1475 (−C–N−); 1393, 1390, 1387 ((CH3)3C−); 1366, 1362, 1358 (CH3−).

##### 6-(Bis(2,6-dimethylphenyl)methyl)-3′,6′-di-*tert*-butyl-9-ethyl-9H-3,9′-bicarbazole radical (Compound **3**)

Yield of purple powder is of 13% (0.047 g). IR ν_max_ (KBr tablet): 3006 (−C–H Ar), 2970, 2968, 2962 (−C–H aliph.); 1483, 1472, 1468 (−C–N−); 1378, 1372, 1370 ((CH3)3C−); 1426, 1418, 1410 (CH3−).

##### 10-(6-(Bis(2,4-dimethylphenyl)methyl)-9-ethyl-9H-carbazol-3-yl)-2,7-di-*tert*-butyl-9,9-dimethyl-9,10-dihydroacridine radical (Compound **4**)

Yield of pale yellow powder is of 24% (0.080 g). IR ν_max_ (KBr tablet): 3015 (−C–H Ar), 2959, 2951 (−C–H aliph.); 1450, 1447, 1440 (−C–N−); 1392, 1342 ((CH3)3C−); 1387, 1366 (CH3−).

##### 10-(6-Benzhydryl-9-ethyl-9H-carbazol-3-yl)-2,7-di-*tert*-butyl-9,9-dimethyl-9,10-dihydroacridine Radical (Compound **5**)

Yield of pale-yellow powder was 30% (0.105 g). IR ν_max_ (KBr tablet): 3025 (−C–H Ar), 2973, 2971, 2965 (−C–H aliph.); 1447, 1440, 1433 (−C–N−); 1315, 1309, 1297 ((CH3)3C−); 1336, 1331, 1328 (CH3−).

##### 10-(6-(Bis(2,6-dimethylphenyl)methyl)-9-ethyl-9H-carbazol-3-yl)-2,7-di-*tert*-butyl-9,9-dimethyl-9,10-dihydroacridine Radical (Compound **6**)

Yield of dark yellow powder is of 17% (0.035 g). IR ν_max_ (KBr tablet): 3006 (−C–H Ar), 2961, 2958, 2955 (−C–H aliph.); 1463, 1460, 1446 (−C–N−); 1352, 1348, 1337 ((CH3)3C−); 1377, 1374, 1361 (CH3−).

## Supporting Information Available

Figure S1: EPR spectra of stable radicals **1**–**6**. Figure S2: TGA and DSC curves. Figure S3: Cyclic voltammograms. Figure S4: Electron photoemission in air spectra. Figure S5: UV–vis and emission spectra of solid samples. Figure S6: Emission and decay spectra of toluene solution of compound **2**. Figure S7: Emission stability test. Figure S8: Transition orbitals of radicals **2** and **6**. Figure S9: DFT energies and visualized transitions of radicals **2** and **6**. Figure S10: Detailed transitions at higher excited states. Figure S11: Water contact angle measurements. Figure S12: SEM images. Figure S13: XRD patterns. Table S1: Calculated reorganization energies. Figure S2: Calculated coupling and interaction energies. Table S3: Electronic coupling values. Table S4: Interaction energies calculation. Table S5: Photovoltaic parameters of solar cells. Table S6: Contact angle values. Table S7: Fitting parameters of TRPL. (PDF)
