# Supplementary material for: Exploring the Charge-Transport and Optical Characteristics of Organic Doublet Radicals: A Theoretical and Experimental Study with Photovoltaic Applications
Source: ACS Appl Mater Interfaces. 2024 Jul 25;16(31):41230–43. doi: 10.1021/acsami.4c08524 (PMC11310911; doi:10.1021/acsami.4c08524)
Supplement: Supplementary file 1 — am4c08524_si_001.pdf [file am4c08524_si_001.pdf]

Supporting Information for

Exploring the Charge-Transport and Optical Characteristics of Organic Doublet Radicals: A Theoretical and Experimental Study with Photovoltaic Applications

Mariia Stanitska[1], Rasa Keruckiene[1*], Gjergji Sini[2*], Dmytro Volyniuk[1], Arunas Marsalka[3], Zhong-En Shi[4], Chung-Ming Liu[4], Yan-Ru Lin[4], Chih-Ping Chen[4,5]*, Juozas V. Grazulevicius[1*]

[1]*Department of Polymer Chemistry and Technology, Kaunas University of Technology, K. Barsausko St. 59, LT-50254, Kaunas, Lithuania*

[2]*Laboratoire de Physicochimie des Polymères et des Interfaces, CY Paris Cergy Université, EA 2528, 5 mail Gay-Lussac, Cergy-Pontoise Cedex 95031, France*

[3]*Faculty of Physics, Vilnius University, Sauletekio st. 9 -3, LT-10222, Vilnius, Lithuania*

[4]*Department of Materials Engineering, Ming Chi University of Technology, 84 Gunjuan Road, Taishan, New Taipei City 24301, Taiwan, Republic of China*

[5]*College of Engineering, Chang Gung University, Taoyuan City, 33302, Taiwan, Republic of China*

[*] Corresponding authors: Rasa Keruckiene rasa.keruckiene@ktu.lt; G. Sini gjergji.sini@cyu.fr; Chih-Ping Chen cpchen@mail.mcut.edu.tw; Juozas V. Grazulevicius juozas.grazulevicius@ktu.lt

Contents

Figures and tables..........S-2

References..........S-18

Figures and tables

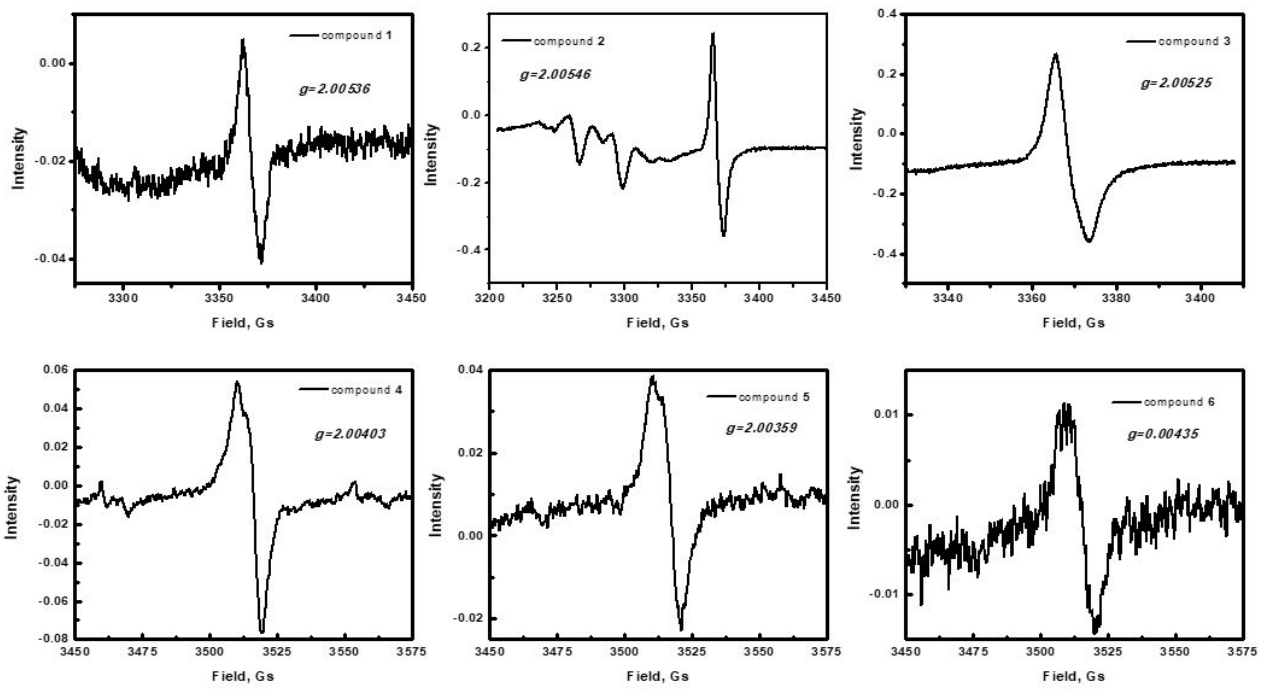

Figure S1. EPR spectra of stable radicals **1–6**.

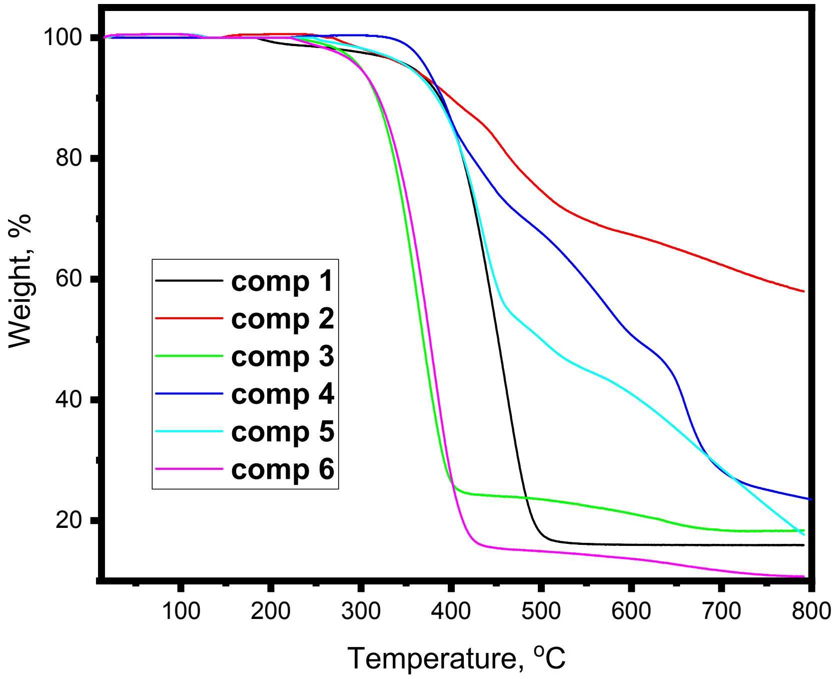

a)

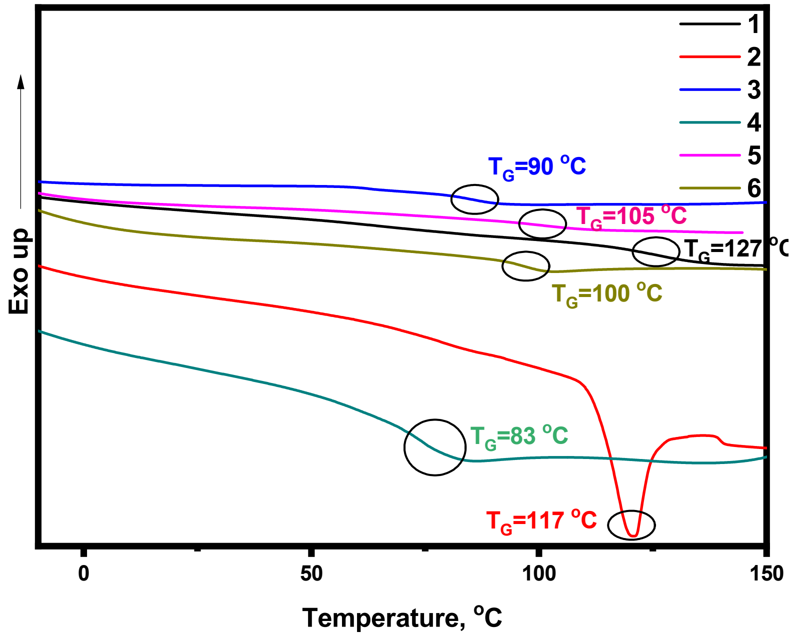

b)

Figure S2. TGA (a) and DSC (b) thermograms of stable radicals **1**–**6**.

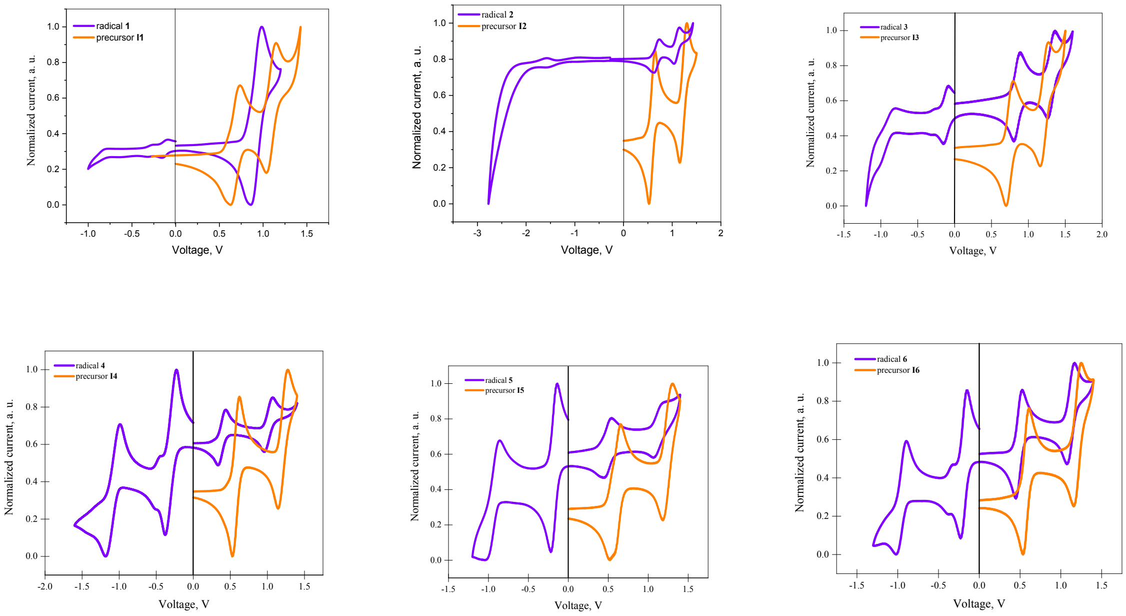

Figure S3. Cyclic voltammograms of intermediate precursors and target stable radicals **1–6**.

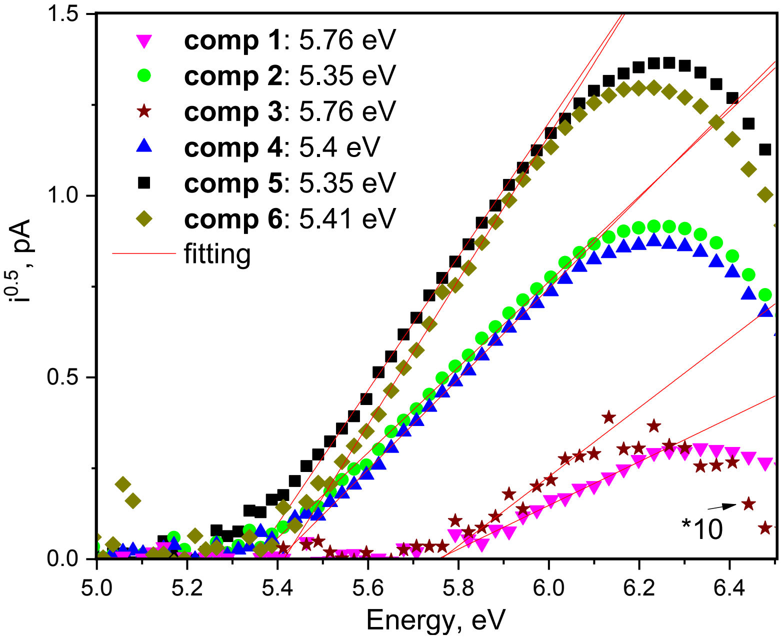

Figure S4. Electron photoemission in air spectra.

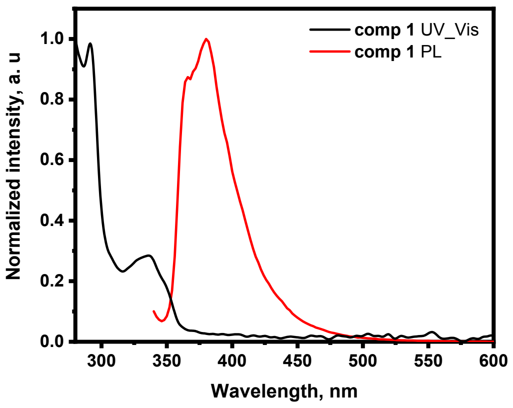
comp 1 UV_Vis
comp 1 PL
Normalized intensity, a. u
Wavelength, nm
0.0
0.2
0.4
0.6
0.8
1.0
300
350
400
450
500
550
600
a)

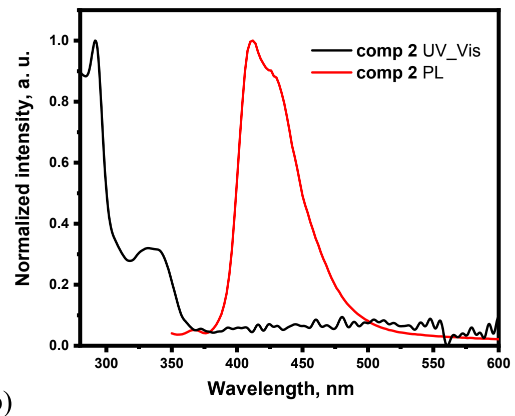
comp 2 UV_Vis
comp 2 PL
Normalized intensity, a. u.
Wavelength, nm
0.0
0.2
0.4
0.6
0.8
1.0
300
350
400
450
500
550
600
b)

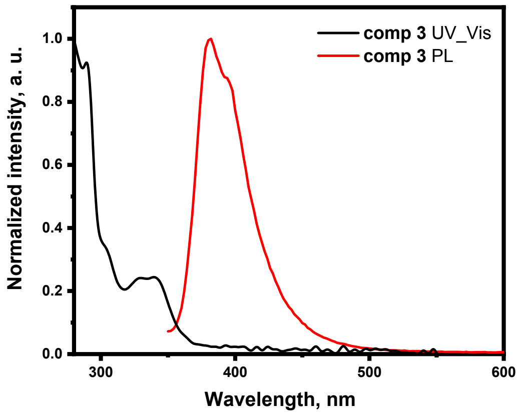
comp 3 UV_Vis
comp 3 PL
Normalized intensity, a. u.
Wavelength, nm
0.0
0.2
0.4
0.6
0.8
1.0
300
400
500
600
c)

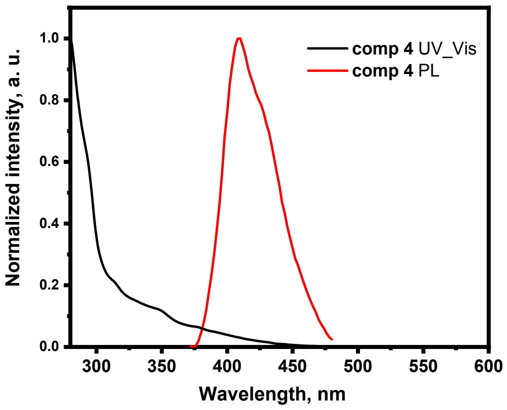
comp 4 UV_Vis
comp 4 PL
Normalized intensity, a. u.
Wavelength, nm
0.0
0.2
0.4
0.6
0.8
1.0
300
350
400
450
500
550
600
d)

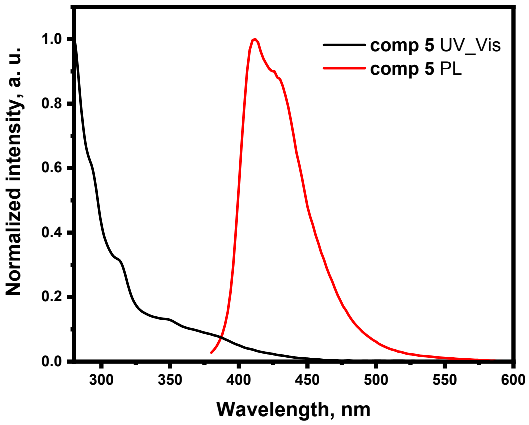
comp 5 UV_Vis
comp 5 PL
Normalized intensity, a. u.
Wavelength, nm
0.0
0.2
0.4
0.6
0.8
1.0
300
350
400
450
500
550
600
e)

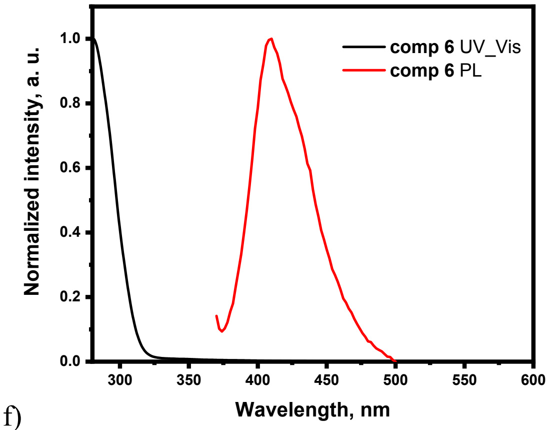
comp 6 UV_Vis
comp 6 PL
Normalized intensity, a. u.
Wavelength, nm
0.0
0.2
0.4
0.6
0.8
1.0
300
350
400
450
500
550
600
f)

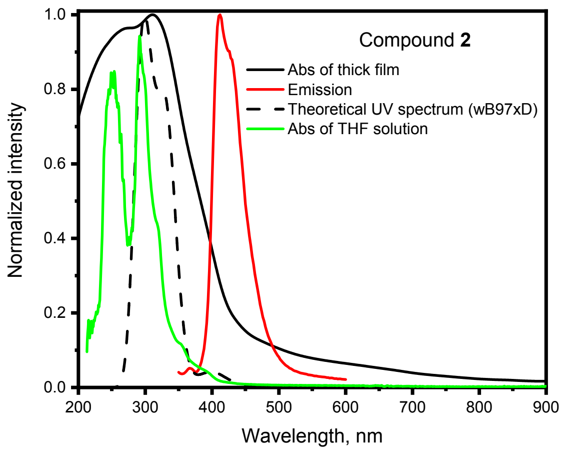

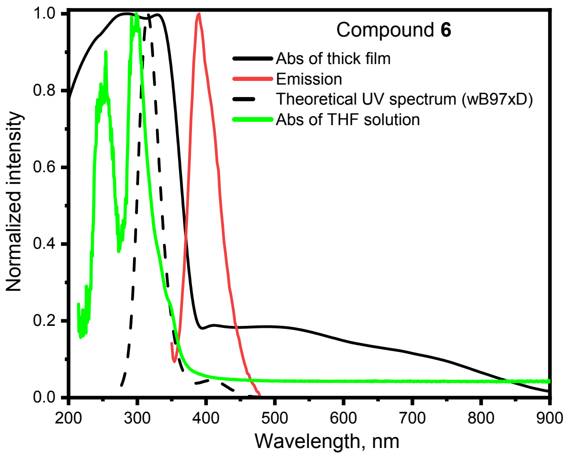

g) h)

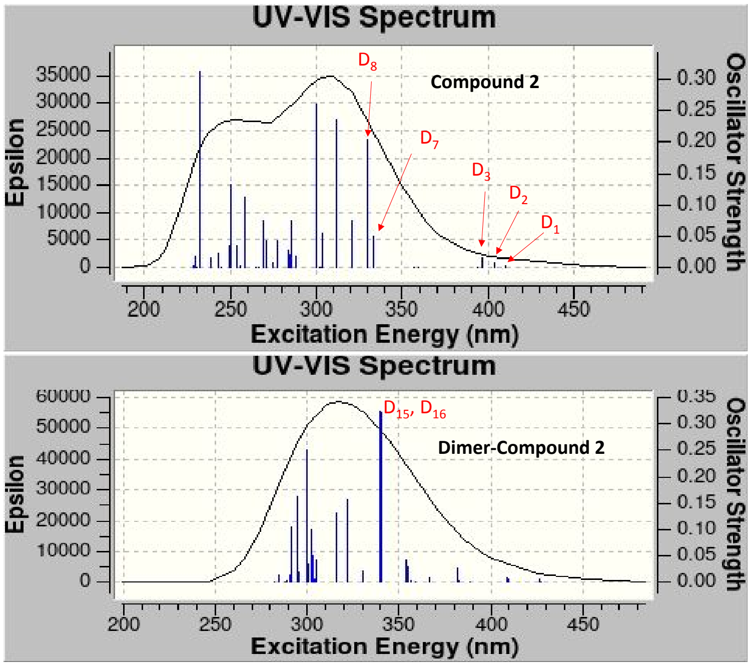

i)

Figure S5. a)-f) UV-Vis and emission spectra of solid films of stable radicals **1**–**6**. g), h) UV-Vis and emission spectra of solid films of stable radicals **2** and **6,** along with their absorption spectra in THF solution; i) theoretical spectra of compound **2** and one of its dimers, both calculated at the ωB97XD/6-31G(d,p) level with the default ω-value.

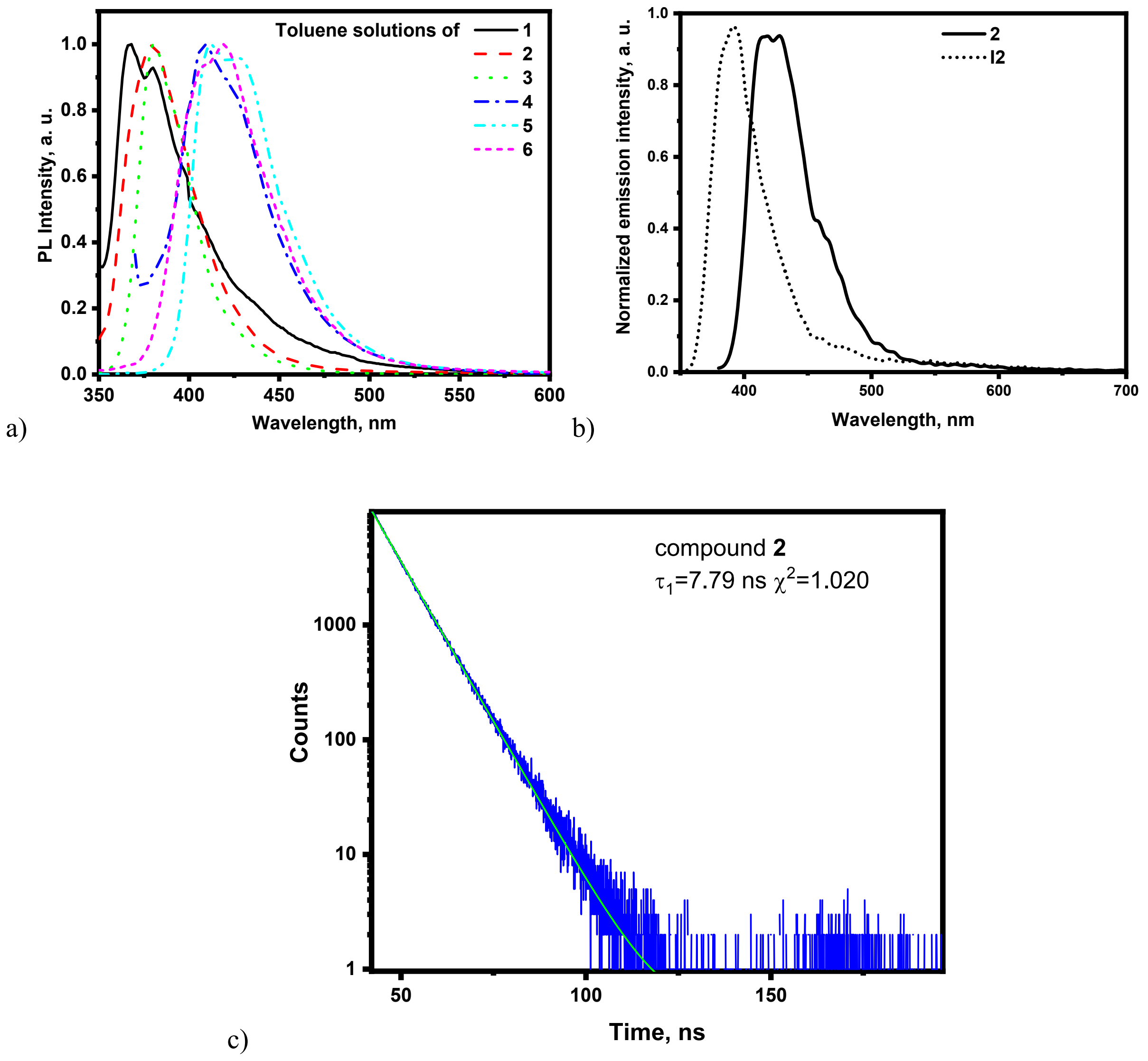

Figure S6. (a) Emission spectra of toluene solutions of stable radicals **1**–**6**; (b) emission spectra of toluene solutions of radical **2** and its precursor **I2**; PL decay curve of radica**l 2** (c).

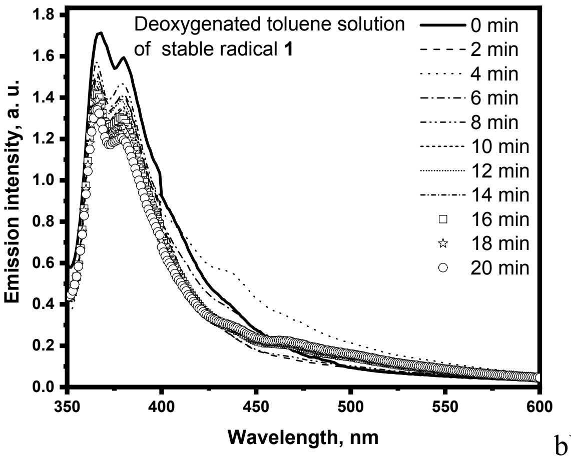

a)

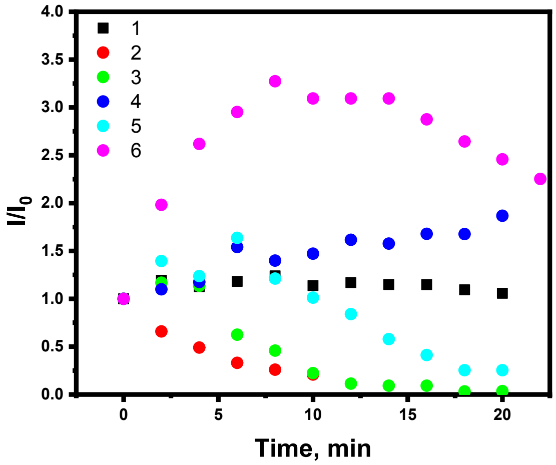

b)

Figure S7. a) Deoxygenated toluene solution of radical **1** at different times; b) Radicals emission stability dependence on time.

Compound **2**

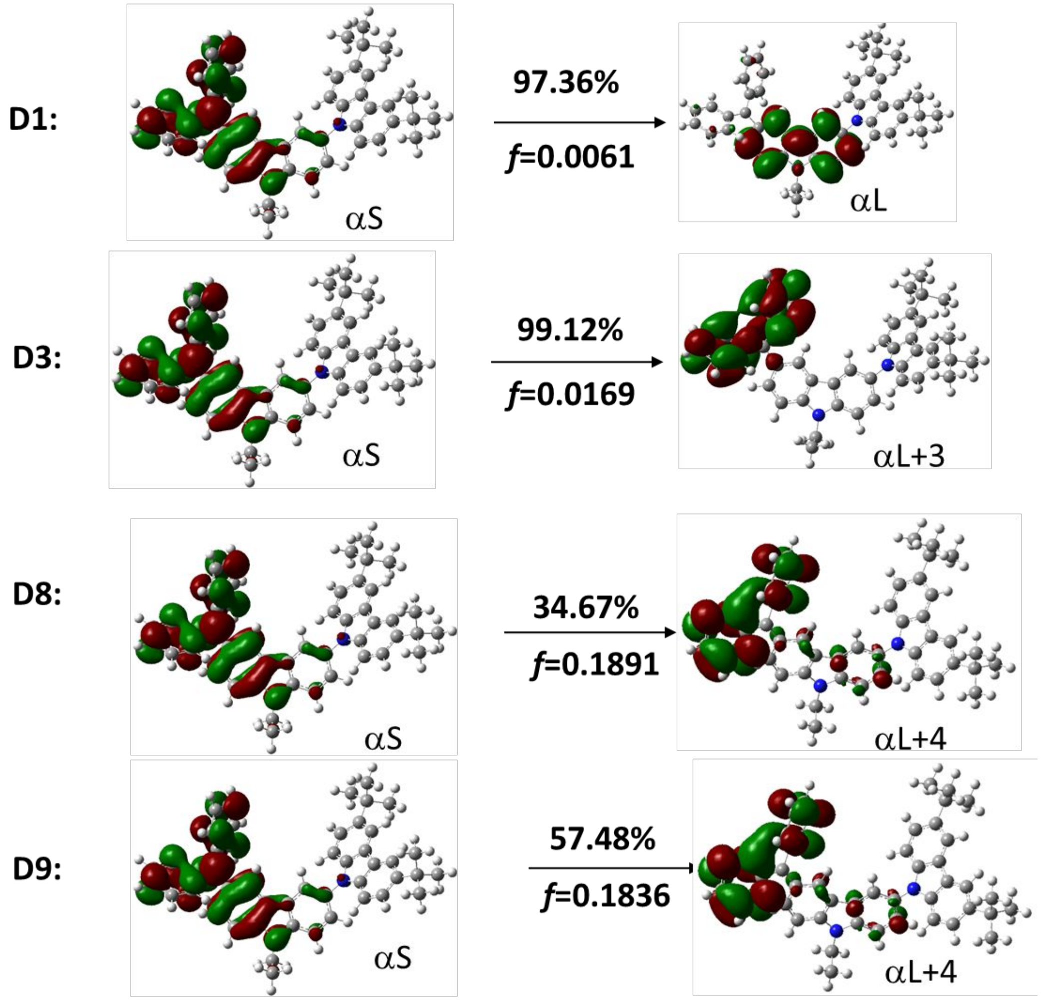
D1:
97.36%
f=0.0061
αS
αL
D3:
99.12%
f=0.0169
αS
αL+3
D8:
34.67%
f=0.1891
αS
αL+4
D9:
57.48%
f=0.1836
αS
αL+4

a)

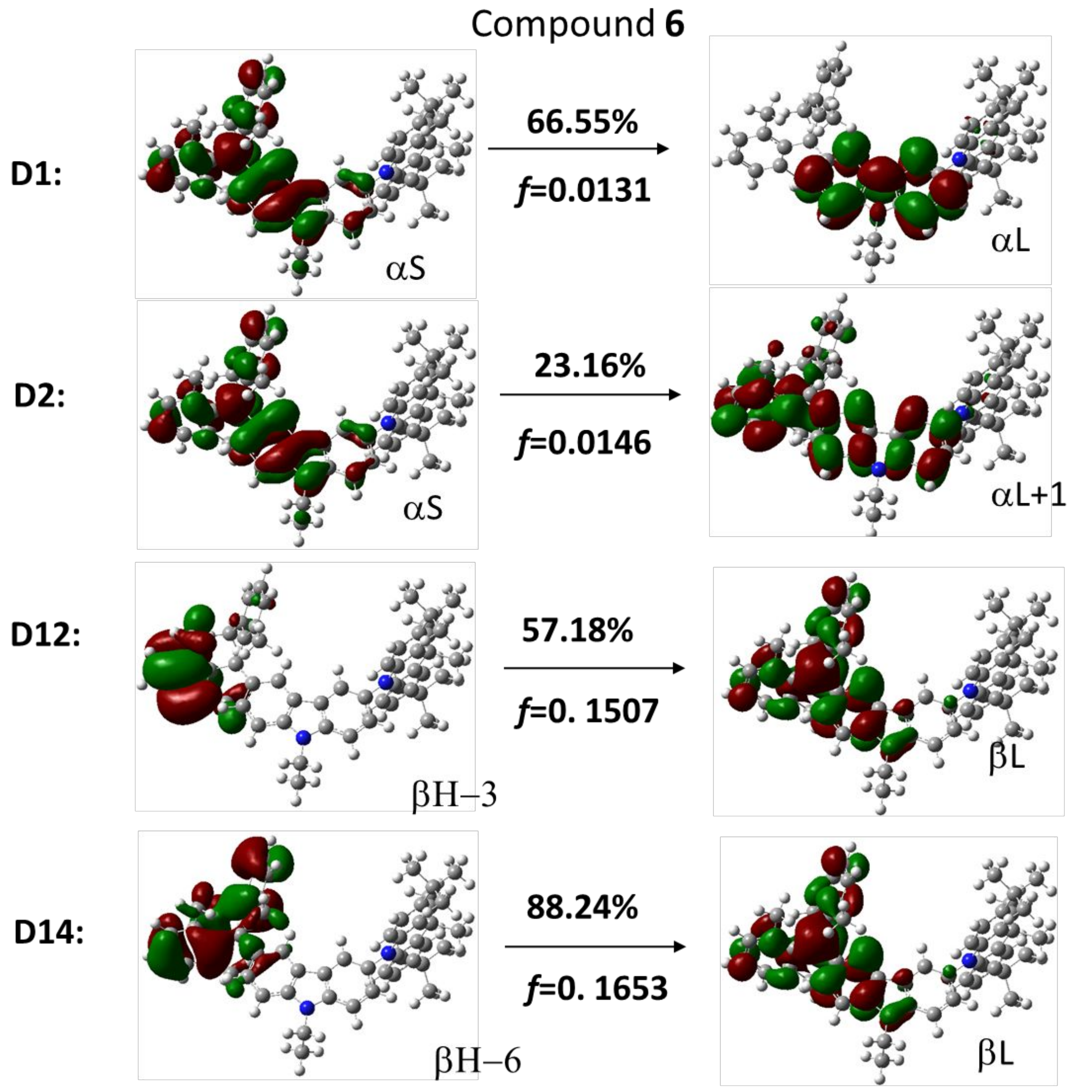

b)

Figure S8. Transition orbitals of radicals **2** (a) and **6** (b)

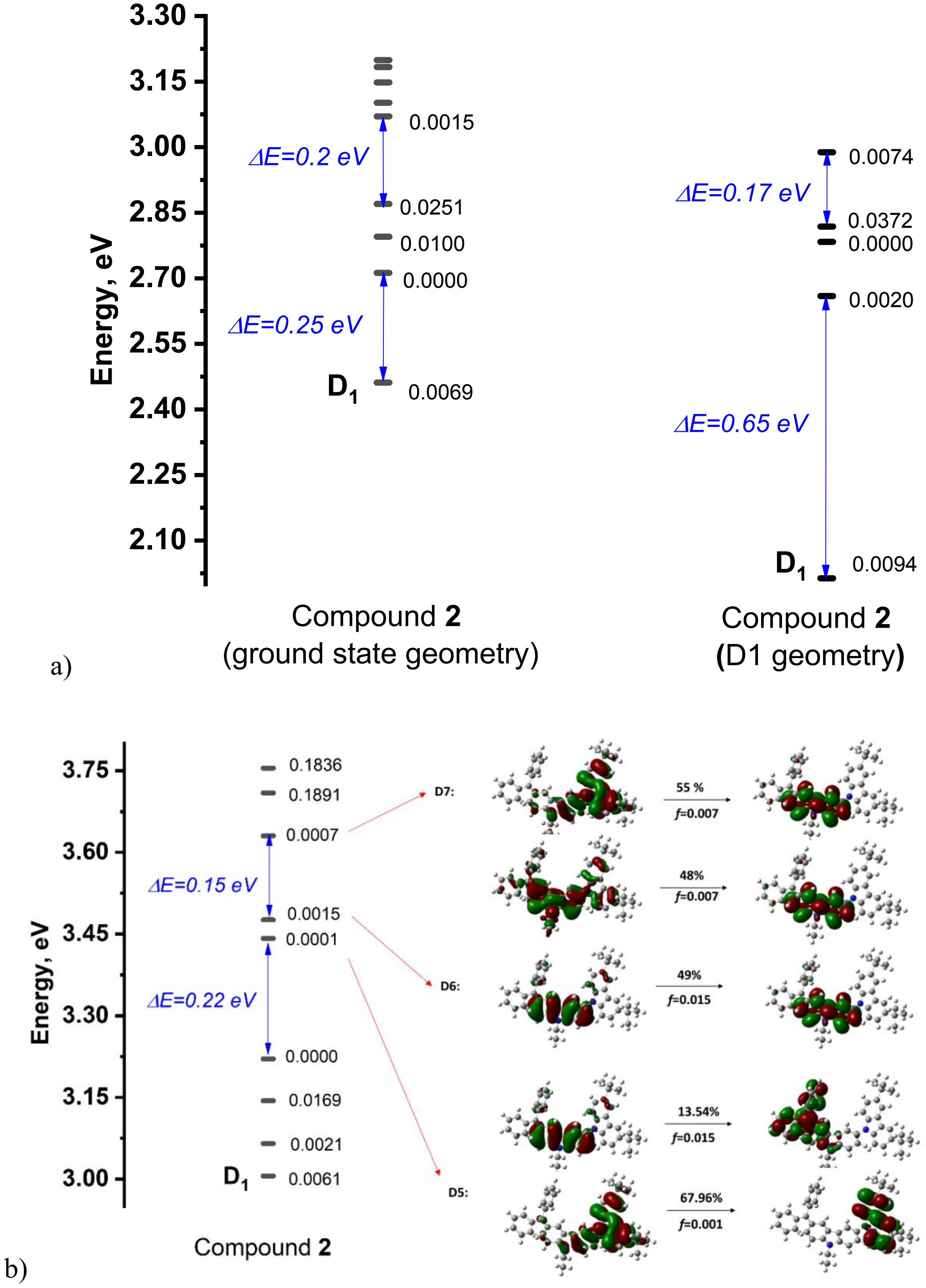

Figure S9. DFT energies of the doublet states of stable radicals **2** and **6** (a); visualized transitions D5,6 and 7 of compound **2** (b).

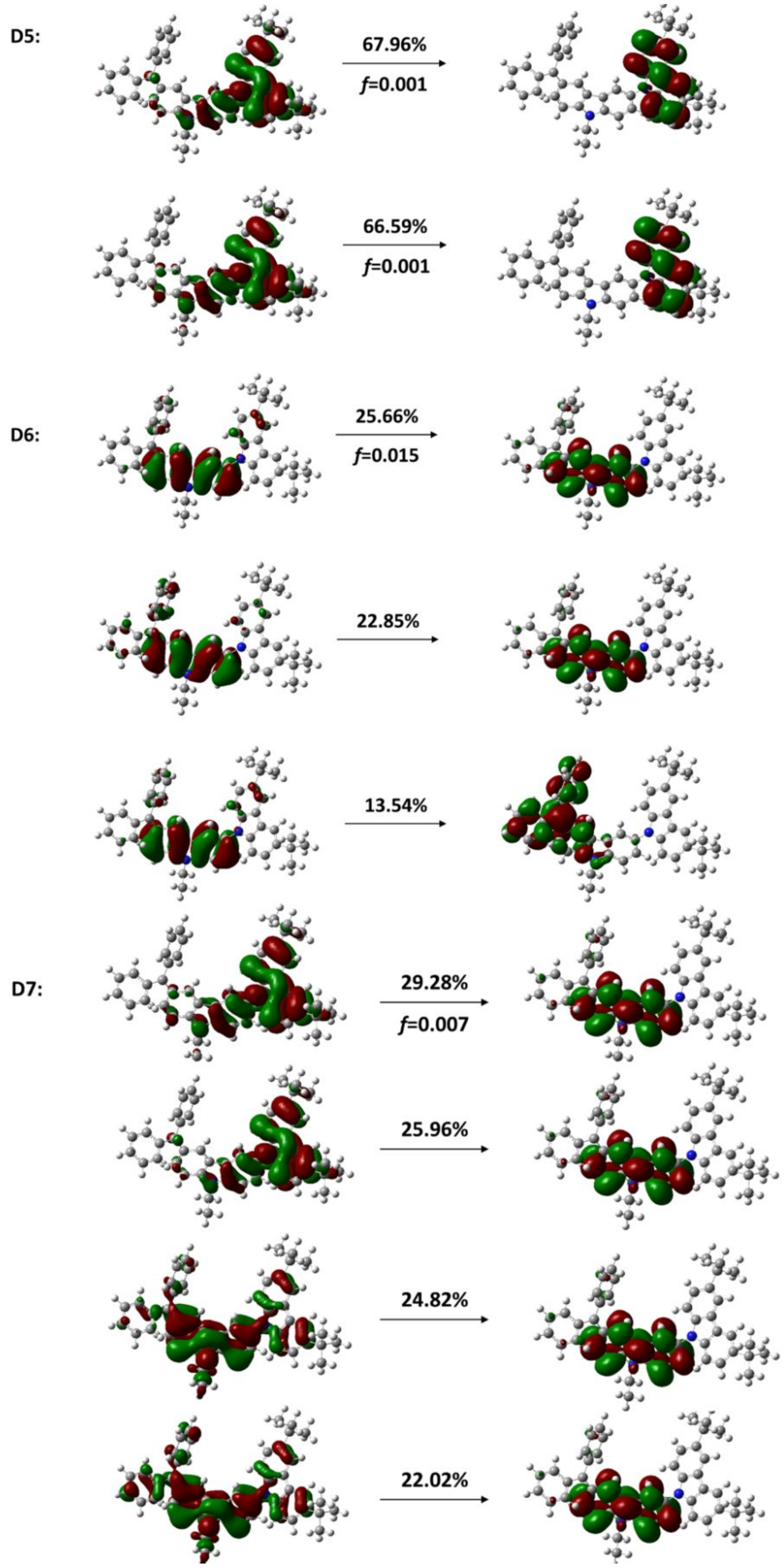

Figure S10. Detailed transitions at the excited states D5, 6 and 7 of compound **2**.

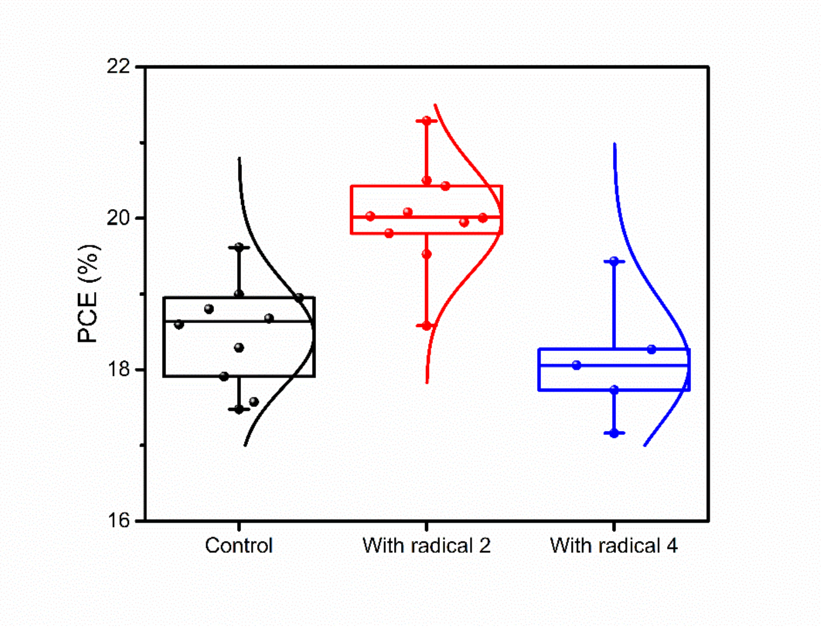

Figure S11. Box plots of PCE of the PSCs with and without radical 2 or 4.

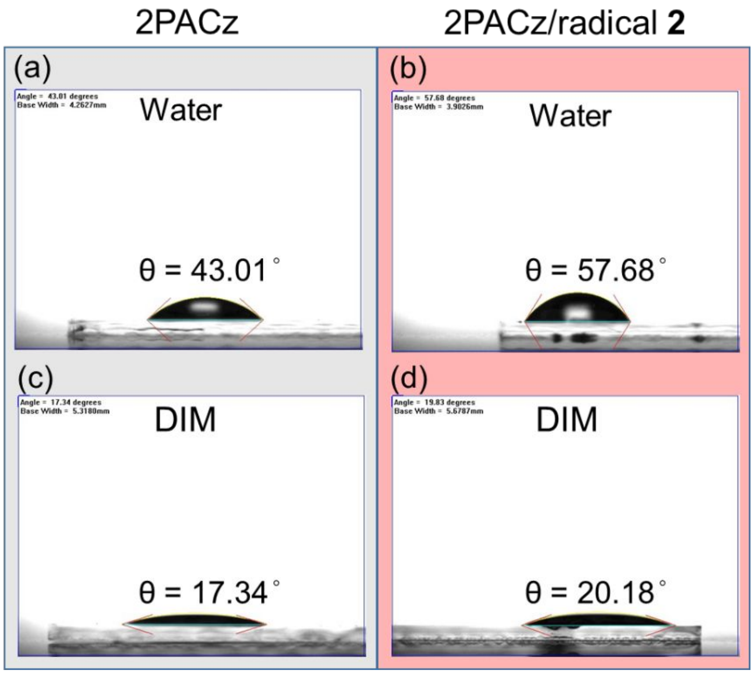

Figure S12. Water contact angle measurements of (a) 2PACz and (b) 2PACz/radical **2**. DIM contact angle of (c) 2PACz and (d) 2PACz/radical **2**.

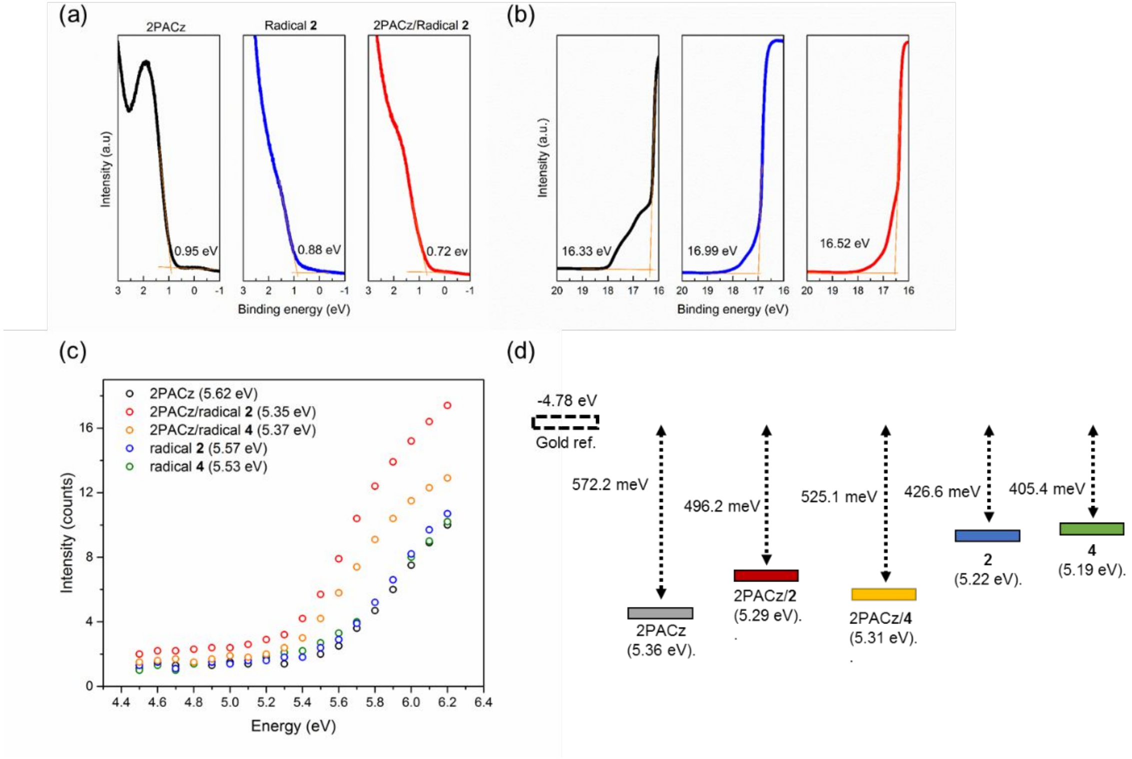

Figure S13. (a) and (b)UPS spectra of 2PACz, radical **2**, and 2PACz/radical **2**. (c) PESA and (d) KP results of HSLs.

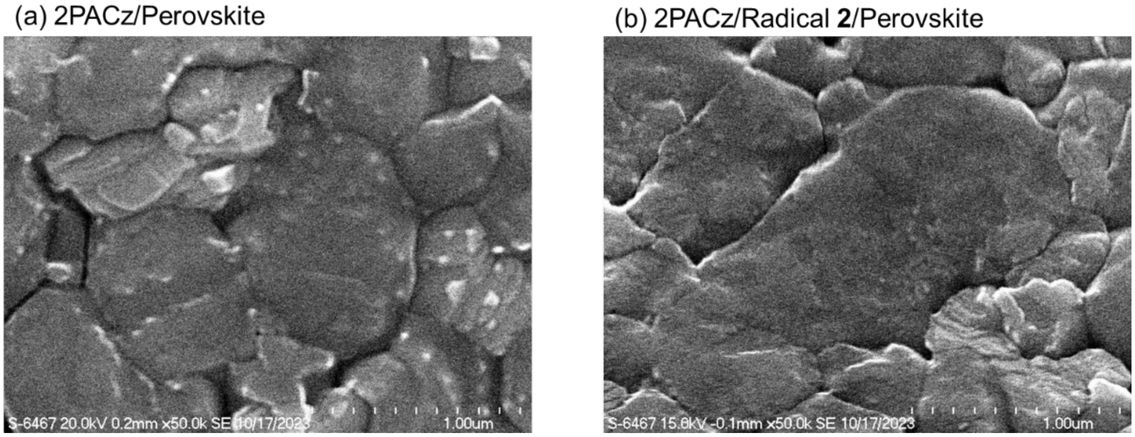

Figure S14. SEM images of (a) 2PACz/Perovskite and (b) 2PACz/radical **2**/Perovskite.

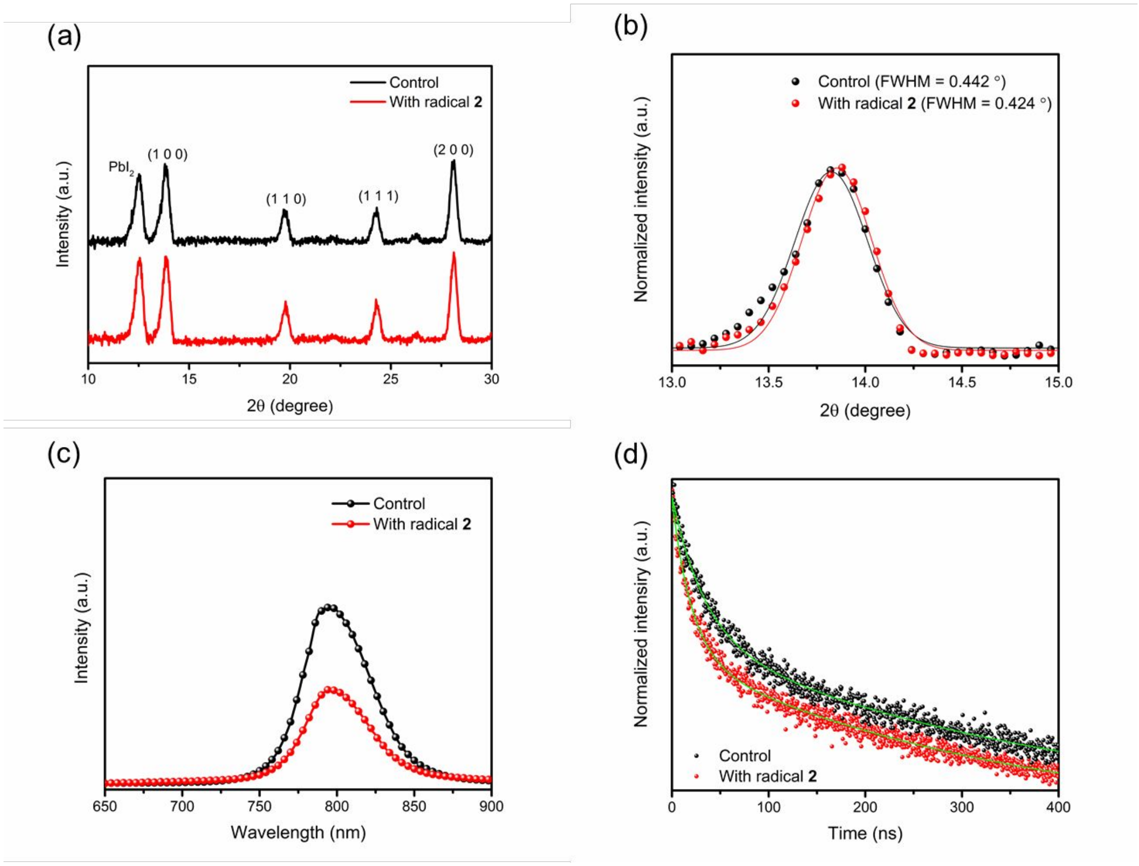

Figure S15. (a) XRD patterns, (b) Narrow XRD signals at a plane of (1 0 0), (c) PL and (d) TRPL of control film (2PACz/Perovskite) and film incorporating with radical **2** (2PACz/radical **2**/Perovskite).

Table S1. Calculated reorganization energies.

**Comp**	**λ+, eV**	**λ-, eV**
**1**	0.2418	0.1934
**2**	0.2389	0.1613
**3**	0.2660	0.2170
**4**	0.2209	0.1890
**5**	0.2492	0.2375

**6**	0.2456	0.2119

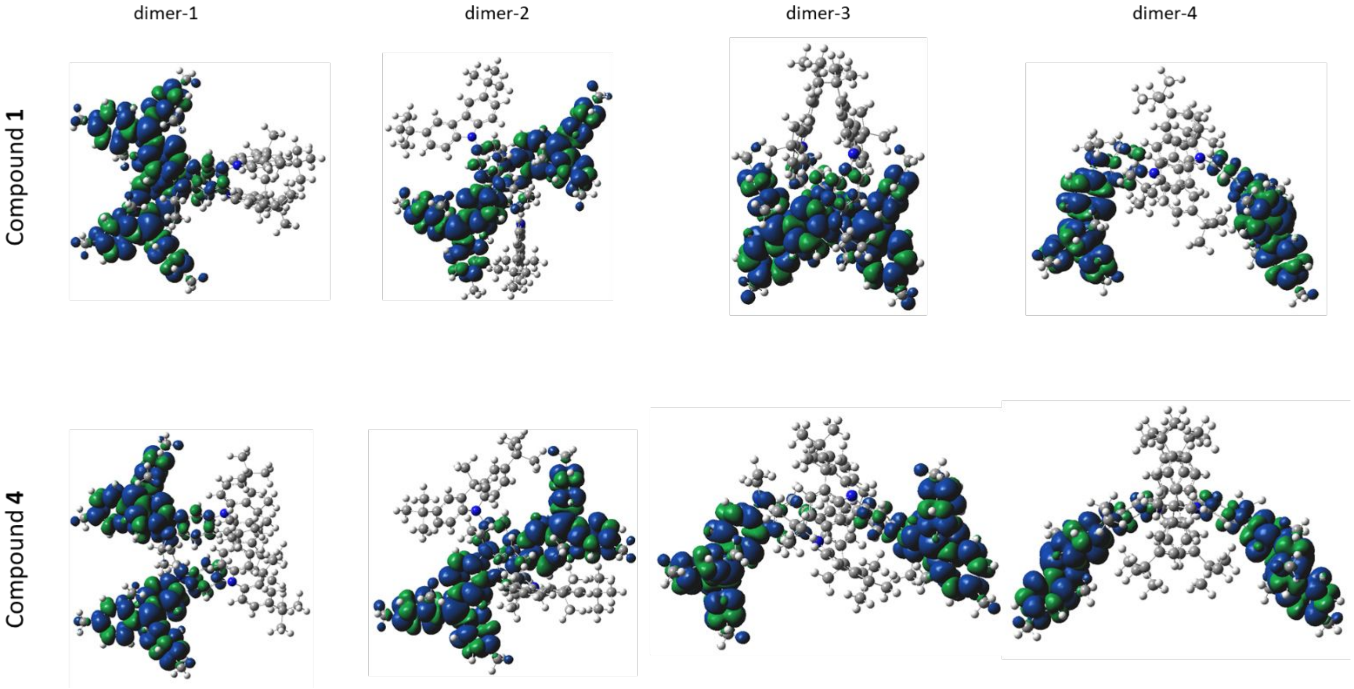

Figure S16. Dimers of stable radicals **1** and **4.**

Table S2. Calculated coupling and interaction energies of dimers of radicals **1** and **4**.

	Radical **1**	Radical **4**
Coupling S-S a (meV)	11.3	3.8
Coupling L-L b (meV)	29.3	18
Interaction en. (kcal/mol)	27	28.7
Standard deviation, eV (SOMO/LUMO)	0.048/0.079	0.046/0.052

Table S3. Electronic coupling values

		Electronic Couplings																	
	dimer	alpha, meV									Beta, meV								
		H-H	H-S	H-L	S-H	S-S	S-L	L-H	L-S	L-L	H-H	H-S	H-L	S-H	S-S	S-L	L-H	L-S	L-L
**comp4**	1	1,6	7,0	16,8	7,4	2,2	8,2	16,5	6,3	54,2	1,6	7,0	15,3	7,5	4,4	9,5	14,9	7,3	50,2
	2	9,1	8,7	29,9	22,2	9,2	41,5	16,3	26,5	27,4	8,3	5,3	19,9	33,8	10,9	22,5	12,8	19,5	14,1
	3	2,1	1,8	3,8	5,4	3,8	0,2	1,3	0,01	0,4	2,1	1,8	3,7	1,2	0,4	0,2	1,3	0,2	0,5
	4	23,0	3,0	16,3	3,4	0,1	0,5	16,4	0,3	6,3	23,0	32,3	15,7	3,7	0,9	0,6	15,7	0,4	7,2
**comp1**	1	30,8	10,1	7,7	10,3	12,6	31,1	9,2	27,8	64,6	35,1	6,7	14,7	7,3	10,3	15,4	16,1	11,4	65,0
	2	6,7	15,4	32,5	0,6	3,1	15,0	45,5	51,3	40,0	8,1	26,0	30,3	2,4	6,1	13,3	35,9	38,2	25,5
	3	11,9	11,6	4,9	8,6	27,5	23,3	6,3	11,2	9,4	14,9	0,6	8,7	4,4	31,4	23,0	8,4	12,9	20,4
	4	57,8	10,5	22,3	9,6	1,9	5,2	21,9	4,9	5,1	55,9	7,9	22,6	6,0	0,9	4,2	22,3	3,9	6,1

Table S4. Interaction energies

	**comp1-dim1**	**comp1-dim2**	**comp1-dim3**	**comp1-dim4**	Average	**comp4-dim1**	**comp4-dim2**	**comp4-dim3**	**comp4-dim4**	Average
E (dimer) neutral	-4168.774324	-4168.767384	-4168.811068	-4168.775572		-4404.588539	-4404.584896	-4404.629707	-4404.581560	
	0.0	4.4	-23.1	-0.8		0.0	2.3	-25.8	4.4	
ZPE	1.829719	1.827887	1.82938	1.829227		1.999366	1.999283	2.001906	1.99994	
BSSE	0.01349398	0.010796949	0.021488657	0.0125827		0.011409907	0.011951086	0.02301533	0.012657809	
	8.5	6.8	13.5			7.2	7.5	14.4	7.9	
E (monomer) neutral	-2084.359078	-2084.359078	-2084.359078	-2084.359078		-2202.265518	-2202.265518	-2202.265518	-2202.265518	
ZPE	0.909839	0.909839	0.909839	0.909839		0.997739	0.997739	0.997739	0.997739	
	6.3	5.2	6.1	6.0		2.4	2.4	4.0	2.8	
Int En	-35.2	-30.9	-58.3	-36.0	**-40**	-36.1	-33.8	-61.9	-31.7	**-41**
Int En + BSSE										
Int En + ZPE+BSSE	-20	-19	-39	-30	**-27.1**	-26.5	-24	-43	-21.0	**-28.7**

Table S5. Photovoltaic parameters of devices with radical **4**. The parameters of the best devices are listed in brackets

Device	J_{SC} (mA/cm^2)	V_{OC} (V)	FF (%)	PCE (%)
With radical **4**	21.31 ± 0.57 (22.42)	1.07 ± 0.01 (1.08)	79.82 ± 0.60 (80.3)	18.13 ± 0.75 (19.43)

Table S6. Photovoltaic parameters of devices with different scan direction.

Device	Scan	J_{SC} (mA/cm^2)	V_{OC} (V)	FF (%)	PCE (%)	HI (%)
Control	Reverse	22.65	1.02	80.4	18.57	2.60
	Forward	22.58	1.00	78.1	17.63	
With radical **2**	Reverse	22.39	1.12	79.7	19.99	1.14
	Forward	22.39	1.12	77.8	19.54	

Table S7. Contact angles and surface energies of 2PACz and 2PACz/radical **2**.

Film	θ_{water} (°)	θ_{DIM} (°)	γ_{polar} (mN m^{-1})	$\gamma_{dispersive}$ (mN m^{-1})	γ_{total} (mN m^{-1})
2PACz	43.36	17.66	24.17	48.46	72.63
2PACz/radical **2**	57.58	20.18	17.43	47.77	65.20

Table S8. Fitting parameters of the TRPL for control film and film incorporating with radical **2**.

Sample	$\tau_{average}$ (ns)	A_1 (%)	τ_1 (ns)	A_2 (%)	τ_2 (ns)
Control	243.50	41.27	33.81	58.73	390.86
With radical **2**	200.60	44.68	20.73	55.32	345.89

References

(1) Gritzner, G.; Kuta, J. Recommendations on Reporting Electrode Potentials in Nonaqueous Solvents (Recommendations 1983). *Pure and Applied Chemistry* **1984**, *56* (4), 461–466. https://doi.org/10.1351/pac198456040461.

(2) Riddell, N.; Jin, U.-H.; Safe, S.; Cheng, Y.; Chittim, B.; Konstantinov, A.; Parette, R.; Pena-Abaurrea, M.; Reiner, E. J.; Poirier, D.; Stefanac, T.; McAlees, A. J.; McCrindle, R. Characterization and Biological Potency of Mono- to Tetra-Halogenated Carbazoles. *Environ Sci Technol* **2015**, *49* (17), 10658–10666. https://doi.org/10.1021/ACS.EST.5B02751.

(3) Fu, B.; Dong, X.; Yu, X.; Zhang, Z.; Sun, L.; Zhu, W.; Liang, X.; Xu, H. Meso-Borneol- and Meso-Carbazole-Substituted Porphyrins: Multifunctional Chromophores with Tunable Electronic Structures and Antitumor Activities. *New Journal of Chemistry* **2021**, *45* (4), 2141–2146. https://doi.org/10.1039/D0NJ02954H.

(4) Bezuglyi, M.; Grybauskaite, G.; Bagdziunas, G.; Grazulevicius, J. V. Crystal Structure of 3-Bromo-9-Ethyl-9H-Carbazole. *Acta Crystallogr E Crystallogr Commun* **2015**, *71* (Pt 12), o1067. https://doi.org/10.1107/S2056989015023907.

(5) Shang, R.; Ilies, L.; Nakamura, E. Iron-Catalyzed Ortho C–H Methylation of Aromatics Bearing a Simple Carbonyl Group with Methylaluminum and Tridentate Phosphine Ligand. *J Am Chem Soc* **2016**, *138* (32), 10132–10135. https://doi.org/10.1021/JACS.6B06908.

(6) Bevan, T. W.; Francis-Taylor, J.; Wong, H.; Northcote, P. T.; Harvey, J. E. A Colourful Azulene-Based Protecting Group for Carboxylic Acids. *Tetrahedron* **2018**, *74* (24), 2942–2955. https://doi.org/10.1016/J.TET.2018.04.066.

(7) Ai, X.; Chen, Y.; Feng, Y.; Li, F. A Stable Room-Temperature Luminescent Biphenylmethyl Radical. *Angewandte Chemie International Edition* **2018**, *57* (11), 2869–2873. https://doi.org/10.1002/ANIE.201713321.
